# Design Principles of the Yeast G1/S Switch

**DOI:** 10.1371/journal.pbio.1001673

**Published:** 2013-10-01

**Authors:** Xiaojing Yang, Kai-Yeung Lau, Volkan Sevim, Chao Tang

**Affiliations:** 1 Center for Quantitative Biology and Peking-Tsinghua Center for Life Sciences, Peking University, Beijing, China; 2 Department of Bioengineering and Therapeutic Sciences, and Center for Systems and Synthetic Biology, University of California, San Francisco, California, United States of America; Wellcome Trust/Cancer Research UK Institute, United Kingdom

## Abstract

A hallmark of the G1/S transition in budding yeast cell cycle is the proteolytic degradation of the B-type cyclin-Cdk stoichiometric inhibitor Sic1. Deleting *SIC1* or altering Sic1 degradation dynamics increases genomic instability. Certain key facts about the parts of the G1/S circuitry are established: phosphorylation of Sic1 on multiple sites is necessary for its destruction, and both the upstream kinase Cln1/2-Cdk1 and the downstream kinase Clb5/6-Cdk1 can phosphorylate Sic1 *in vitro* with varied specificity, cooperativity, and processivity. However, how the system works as a whole is still controversial due to discrepancies between *in vitro*, *in vivo*, and theoretical studies. Here, by monitoring Sic1 destruction in real time in individual cells under various perturbations to the system, we provide a clear picture of how the circuitry functions as a switch *in vivo*. We show that Cln1/2-Cdk1 sets the proper timing of Sic1 destruction, but does not contribute to its destruction speed; thus, it acts only as a trigger. Sic1's inhibition target Clb5/6-Cdk1 controls the speed of Sic1 destruction through a double-negative feedback loop, ensuring a robust all-or-none transition for Clb5/6-Cdk1 activity. Furthermore, we demonstrate that the degradation of a single-phosphosite mutant of Sic1 is rapid and switch-like, just as the wild-type form. Our mathematical model confirms our understanding of the circuit and demonstrates that the substrate sharing between the two kinases is not a redundancy but a part of the design to overcome the trade-off between the timing and sharpness of Sic1 degradation. Our study provides direct mechanistic insight into the design features underlying the yeast G1/S switch.

## Abstract 2

Single-cell microscopy and computational modeling offer novel mechanistic insight into the G1/S switch that initiates DNA replication in budding yeast, revealing a Clb5/6-Cdk1 and Sic1 feedback loop and new rules of biochemical circuit design.

## Author Summary

In eukaryotic organisms, genome replication starts simultaneously from many sites on the DNA, called origins of replication. In budding yeast, these origins are activated by a kinase, Clb5/6-Cdk1. Until the start of S-phase, when the replication origins are activated, this kinase is kept inactive by an inhibitor, Sic1, which has multiple phosphorylation sites. Sic1 phosphorylation at the onset of S-phase leads to its rapid destruction, unleashing a stockpile of Clb5/6-Cdk1. Here, we show using live-cell fluorescent microscopy that Clb5/6-Cdk1 phosphorylation of Sic1 creates a feedback loop that functions as a switch. Our experiments reveal that the feedback loop shields Sic1 destruction from molecular fluctuations and environmental variability, ensuring that the switch flips decisively. We also demonstrate that a multisite phosphorylation scheme is not required for rapid Sic1 destruction. Sic1 can also be phosphorylated by another kinase, called Cln1/2-Cdk1. We demonstrate that this seemingly redundant interaction is responsible for robust timing of Sic1 destruction. Our experiments and mathematical model identify the contribution of each component to the function of this biochemical circuit.

## Introduction

**Figure 1:**
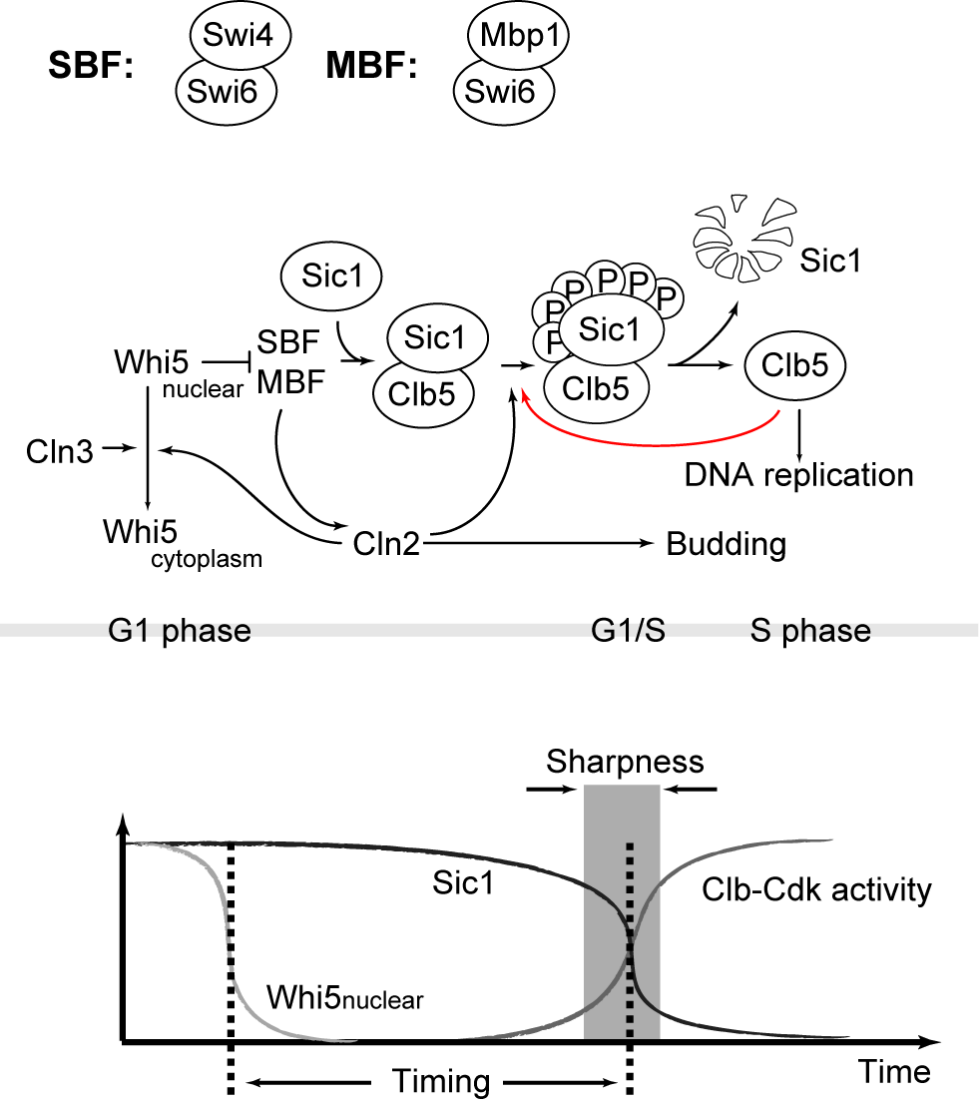
The molecular network controlling the G1/S transition in budding yeast. Black lines represent the current understanding; the role of the red line is discussed in the article.

In the cell cycle of the budding yeast *Saccharomyces cerevisiae*, DNA replication initiation is driven by a sharp rise of Clb5/6-Cdk1 activity [1]–[3]. As the cell passes Start [4],[5], the commitment to the next round of cell division, the transcriptional inhibitor Whi5, is phosphorylated and then excluded from the nucleus [6],[7], and two transcription factor complexes Swi4/Swi6 (SBF) [8] and Mbp1/Swi6 (MBF) [9] are activated. SBF and MBF transcribe about 200 G1/S genes including the G1 cyclins (*CLN1* and *CLN2*) and the S cyclins (*CLB5* and *CLB6*) [10],[11]. Unlike Cln1/2-Cdk1, which is the major driver of cell cycle progression in the late G1 phase by phosphorylating many G1/S targets [12], Clb5/6-Cdk1 is rendered inactive throughout G1 phase by the inhibitor Sic1 until Sic1 is phosphorylated and degraded (Figure 1) [13],[14]. The timing and the speed of Sic1 destruction determine the activation profile of Clb5/6-Cdk1 activity. Strains with either *SIC1* deleted or altered Sic1 degradation dynamics show a significant increase in genomic instability (Figure S4), underlining the importance of Sic1 for a proper S-phase entry [15],[16].

Sic1 has nine consensus CDK phosphorylation sites, and phosphorylation on multiple sites is required for the SCF ubiquitin ligase F-box protein Cdc4 to efficiently recognize Sic1, thereby targeting it for degradation by the 26S proteasome [14],[15],[17]. Both Cln2-Cdk1 and Clb5-Cdk1 can phosphorylate Sic1 efficiently *in vitro* [18],[19], and a recent biochemical study suggested the multisite phosphorylation is carried out in a processive cascade fashion [18]. Given the *in vitro* observation that Cln2-Cdk1 and Clb5-Cdk1 have different specificity on different phosphorylation sites of Sic1, it was proposed that Sic1 is first phosphorylated by Cln2-Cdk1 on a certain site (priming site), facilitating its subsequent phosphorylation by Clb5-Cdk1, eventually leading to its degradation after phosphorylation of the phosphodegrons [18].

However, this interpretation is clouded by extensive circumstantial *in vivo* evidence. Strains lacking *CLN1* and *CLN2* are highly sensitive to *SIC1* gene dosage [20], the DNA replication delay in strains lacking *CLN1* and *CLN2* is Sic1 dependent [21], premature Clb5 expression from the GAL1 promoter does not advance the onset of S phase [2], and deleting *CLB5* and *CLB6* does not affect the normal timing of Sic1 turnover [14],[22]. All *in vivo* observations seem to suggest that it is Cln1/2-Cdk1, but not Clb5/6-Cdk1, who is responsible for the physiological Sic1 destruction. It was even thought that the only nonredundant essential function of the Cln1/2-Cdk1 is to inactivate Sic1 [23]. To date, the only supporting evidence for Clb5/6-Cdk1's contribution to Sic1 destruction *in vivo* is that Sic1 is stable with B-type cyclin inhibition [18]. However, the same experiment performed by another group earlier reached the opposite conclusion [14]. Therefore, whether or not Clb5/6-Cdk1 phosphorylates Sic1 *in vivo* remains a mystery.

In addition, previous studies suggested that the requirement of multisite phosphorylation sets a threshold for CDK activity for the onset of Sic1 degradation, and thus is responsible for a rapid, switch-like destruction of Sic1 [15],[24]. However, a theoretical study showed that while multisite phosphorylation can establish a threshold for kinase activity, the response beyond the threshold is not necessarily switch-like [25]. The same study reported that a switch-like response requires disparities of several orders of magnitude in catalytic efficiencies at different phosphorylation sites. This does not seem to be the case for Sic1 [18].

In this article, by monitoring Sic1 destruction dynamics directly in single cells and in real time under a variety of systemic and environmental perturbations, we resolve the discrepancies mentioned above and provide a clear picture of the G1/S transition in yeast. We investigate the role various components in the G1/S regulatory circuitry play in Sic1 destruction dynamics and dissect the G1/S switch at the core of this circuitry to reveal its underlying design principles.

## Results

### Clb5/6-Cdk1, Not Cln1/2-Cdk1, Plays the Major Role in Controlling the Speed of Sic1 Destruction *in Vivo*

**Figure 2:**
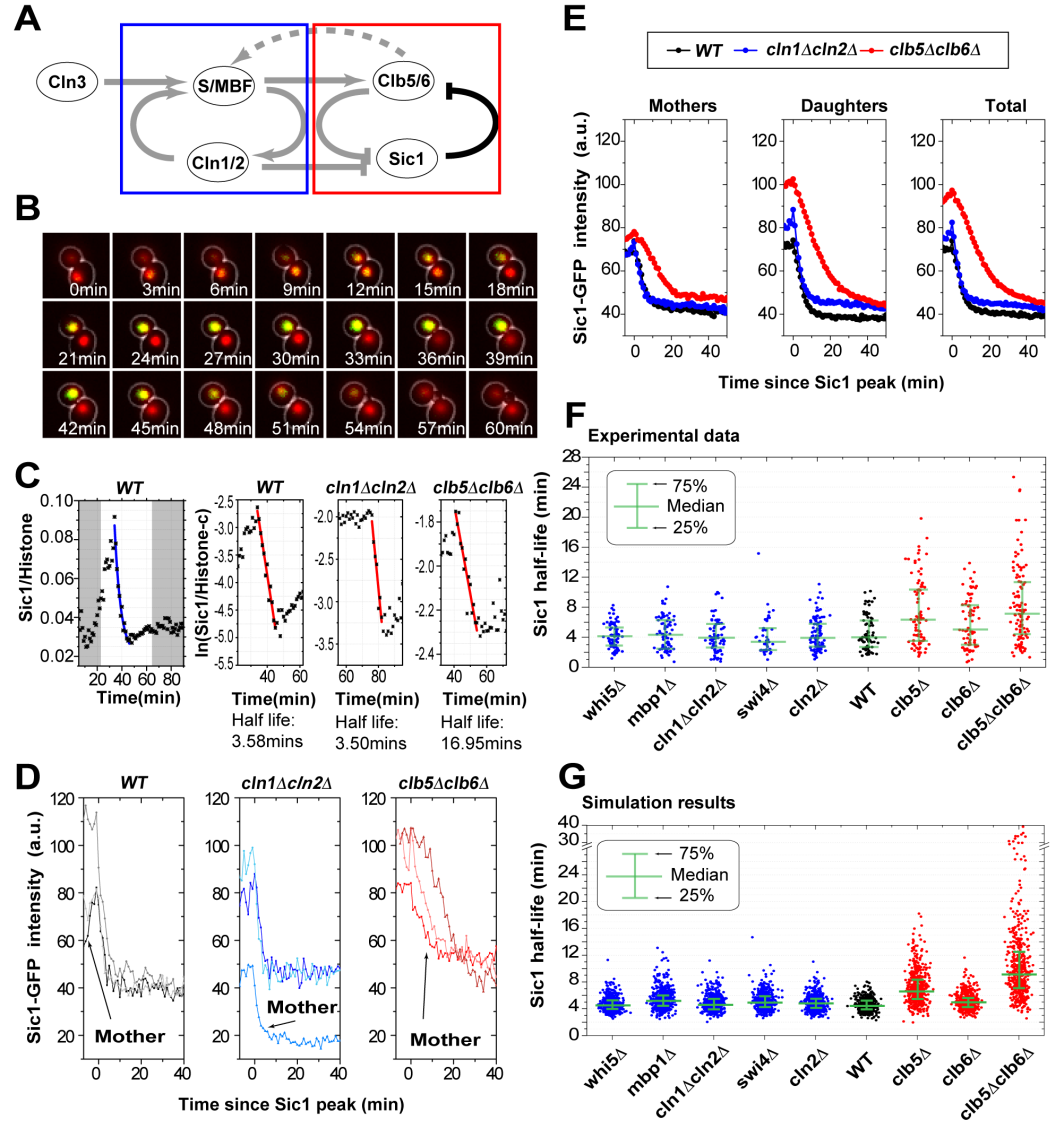
The speed of Sic1 destruction *in vivo* is controlled by Clb5/6-Cdk1. (A) The topology of the network. The parts encircled by the blue and red rectangles are topologically two feedback loops. (B) Combined phase and fluorescence time-course images in wild-type cells (green, the endogenous Sic1; red, histone marker for the nucleus). (C) A sample Sic1-GFP time course used to extract half-life. The leftmost panel is a linear plot for wild-type, with the blue line being a fit to the exponential function *a*exp(−*bt*)+*c*. The three right panels are semi-log plots, with the red line being a fit to ln(*a*)−*bt*. (see Text S1 for details) (D) Sic1 single cell profile samples. The profiles are aligned at the time point of the maximum Sic1 concentration (time interval, 1 min). (E) Average Sic1 profiles for the three strains indicated (the same alignment as in E). (F) Sic1 half-life for wild-type and various deletion strains. (G) Simulation results from a stochastic model of the system. Each data point corresponds to a different realization (see Text S2 for details).

To quantitatively monitor Sic1 degradation dynamics in individual cells and in real time, we tagged the endogenous *SIC1* at the C terminus with a green fluorescent protein (GFP) and used live-cell fluorescence microscopy (Figure 2B). We verified that Sic1-GFP half-life is the same as the endogenous Sic1 (Figure S1A–C). For each cell, we were able to measure the concentration of Sic1 as a function of time and thereby obtained the half-life of Sic1 by fitting our data to an exponential decay function (Figure 2C, Text S1). We observed considerable cell-to-cell variability in Sic1 half-life (Figure 2F), though there is no systematic difference between mother and daughter cells (Figure 2D,E, Figure S1F, Figure S2F–H, and Table S1).

We first deleted one by one each of the components in the circuitry that precedes Clb5/6-Cdk1 activation (blue box in Figure 2A). Perturbations on different components led to the various familiar phenotypes: For example, *swi4Δ* and *cln1Δcln2Δ* prolonged the cell cycle, *whi5Δ* had a smaller cell size, and *cln1Δcln2Δ* delayed budding. However, none of these has a statistically significant effect on Sic1 half-life (Figure 2F; Tables S1 and S2). Surprisingly, even deletion of both *CLN1* and *CLN2* had no effect on the speed of Sic1 destruction (Figure 2D–F; Tables S1 and S2). These findings suggest that none of these components, including Cln1/2, contributes significantly to the speed and variability of Sic1 destruction. This is in stark contrast with the current model suggested by earlier studies, in which only Cln2-Cdk1 is responsible for switch-like destruction of Sic1 [15],[24].

We next deleted *CLB5* and *CLB6*. In this case, large effects were observed on both the median and the variability of Sic1 half-life (Figure 2D–F; Tables S1 and S2). The median value increased to *τ_clb5Δ_* = 6.35 min and *τ_clb5Δclb6Δ_* = 7.14 min in *clb5Δ* and *clb5Δclb6Δ* strains, respectively, compared to *τ*_WT = _3.93 min. The variability also increased significantly. These results suggest that Clb5/6-Cdk1 plays a critical role in controlling the speed of Sic1 destruction. The effects of the various gene deletions on Sic1 degradation dynamics were quantitatively captured in a stochastic model of the entire G1/S circuitry (Text S2, Figure 2G, Figure S1G). An important point worth noting is that the slow degradation we observed in *clb5Δclb6Δ* cells suggests that Cln1/2-Cdk1 mediated phosphorylation does not lead to Sic1's fast destruction. This finding argues against the current model, in which Cln1/2-Cdk1 is solely responsible for rapid destruction of Sic1 [15].

The result above is in line with the *in vitro* study that showed that Clb5-Cdk1 is more potent than Cln2-Cdk1 on more phosphorylation sites of Sic1 [18]. Furthermore, it provides direct evidence that a double-negative feedback loop between Clb5/6-Cdk1 and Sic1 is in action (Figure 1 and red box in Figure 2A). Positive feedback loops are capable of generating sharp transitions and are widely implemented in cell fate circuitries [26]–[30]. The observed fast degradation of Sic1 attributed to Clb5/6-Cdk1 can either be due to a higher potency of Clb5/6-Cdk1 on Sic1 phosphorylation or the double-negative feedback loop, or both. Thereby, we next sought to disentangle the contribution of the feedback loop from that of the kinase on Sic1 destruction.

### The Double-Negative Feedback Loop Between Clb5/6-Cdk1 and Sic1 Functions as a Noise Filter to Ensure Robust Fast Destruction of Sic1 in the Face of Genetic and Environmental Perturbations

**Figure 3:**
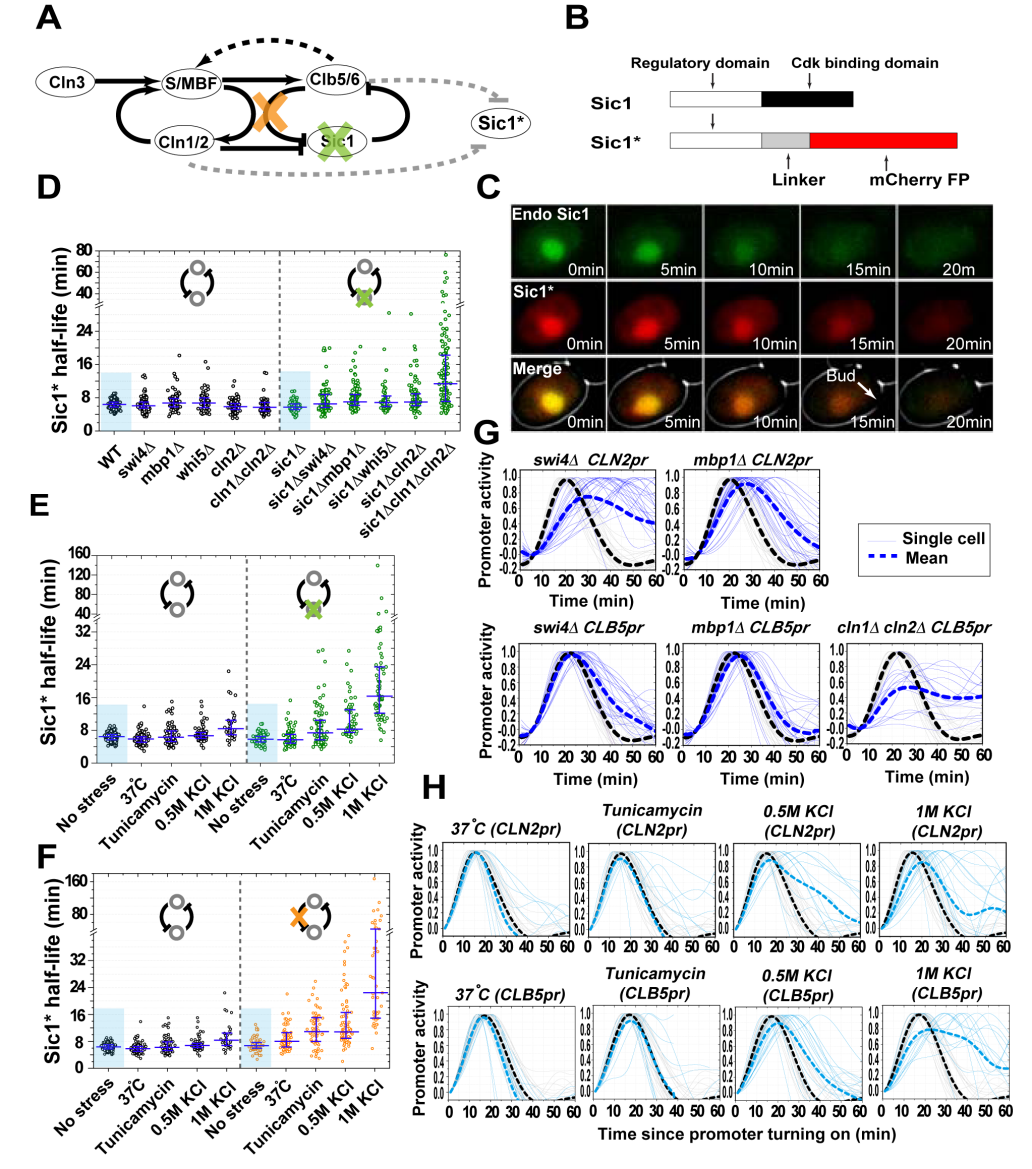
The double-negative feedback loop (DNFBL) between Sic1 and Clb5/6-Cdk1 ensures robustness in Sic1 destruction speed. (A) Black lines, the topology of the G1/S switch. Grey lines, interaction of the reporter Sic1* with the circuit. Sic1* “reports” the destruction dynamics of Sic1. Perturbations to the DNFBL are indicated by the colored crosses. (B) The construction of the reporter Sic1*. (C) Sic1* has the same subcellular localization and the same degradation dynamics as the endogenous Sic1. Shown in the figure is fluorescence time course images of the reporter Sic1*(red) and the endogenous Sic1 (green). (D–F) Sic1* half-life with the DNFBL intact (black dots) and perturbed (colored dots). (D) The DNFBL was perturbed by deleting *SIC1*. The cells were further subjected to genetic perturbations as indicated below the data points. (E) The DNFBL was perturbed by deleting *SIC1*. The cells were further subjected to environmental perturbations as indicated below the data points. (F) The DNFBL was perturbed by deleting the link between Clb5/6-Cdk1 and Sic1, which was accomplished by using the nonphosphorylatable Sic1-0p [44]. The cells were further subjected to environmental perturbations as indicated below the data points. (G–H) The activity of *CLN2* and *CLB5* promoter under various genetic perturbations (G) and environmental stress (H). Each grey line is from an unstressed cell; each colored line is from a stressed cell. Thick dashed lines are averages of the two groups, respectively. The lines were smoothed for clarity.

To investigate the function of the feedback loop, we constructed a Sic1 reporter, designated Sic1*, by fusing the regulatory domain of Sic1 (including all nine CDK phosphorylation sites) [31] to a fluorescent protein (mCherry) and placing it under the control of the *ADH1* constitutive promoter (Figure 3A and 3B). We verified that Sic1* has the same subcellular localization and the same degradation dynamics as the endogenous Sic1 (Figure 3C, Figure S2A) and that Sic1* does not inhibit Clb5/6-Cdk1 (Figure S2B). Thus Sic1* can serve as a reporter of Sic1 destruction dynamics, but due to its lacking of the CDK binding domain, it cannot inhibit Clb5/6-Cdk1.

With the reporter Sic1*, we were able to study the role of the other components in the feedback loop on Sic1 destruction dynamics (Figure 3A and D–F). Interestingly, unlike the deletion of *CLB5/6* (Figure 2F), disabling other components of the double-negative feedback loop resulted in minimal impact on Sic1 degradation speed (compare pairs of data in black and colored dots highlighted by the small rectangles in Figure 3D–F), suggesting that it is Clb5/6-Cdk1, not the feedback loop, that is responsible for Sic1's fast destruction.

However, when the cell is subject to genetic or environmental perturbations and stress, we observed a very different behavior: the absence of the loop resulted in large variations in the degradation speed, suggesting that the feedback loop is necessary for robustly fast Sic1 destruction under perturbations (Figure 3D–F, Tables S3, S4, S5, S6, S7).

These genetic and environmental perturbations led to noisy transcriptional activities in *CLN2* and *CLB5* (Figure 3G and 3H, Table S8), and some perturbations increased the cell cycle period more than 2-fold (Figure S2C). (Notably, in the absence of the loop, the degree of the variability in Sic1 half-life reflects the degree of variability in transcriptional activity of *CLN2* and *CLB5* (Figures 3D–H).) Remarkably, the double-negative feedback loop can buffer the extrinsic noise (cell-to-cell variability) and fluctuations to ensure a robust fast destruction of Sic1.

### The Timing of Sic1 Destruction Is Set by Cln1/2-Cdk1

So far we have established the important role of Clb5/6-Cdk1 and the double-negative feedback loop in Sic1 destruction. On the other hand, the upstream kinase Cln1/2-Cdk1 can phosphorylate Sic1 *in vitro* [14] and, presumably, also contributes to Sic1 degradation *in vivo* as suggested both by previous work [20],[21] and by our experiment with the *clb5Δclb6Δ* strain. We have shown that Cln1/2-Cdk1 is not essential for Sic1's fast destruction. What is then the *in vivo* role, if any, of Cln1/2-Cdk1 here? It is known that in *cln2Δ* strains the onset of the S phase is significantly delayed [21], and recent biochemical study found that Cln2-Cdk1 has a preferred phosphorylation site on Sic1 that could function as a priming site [18].

**Figure 4:**
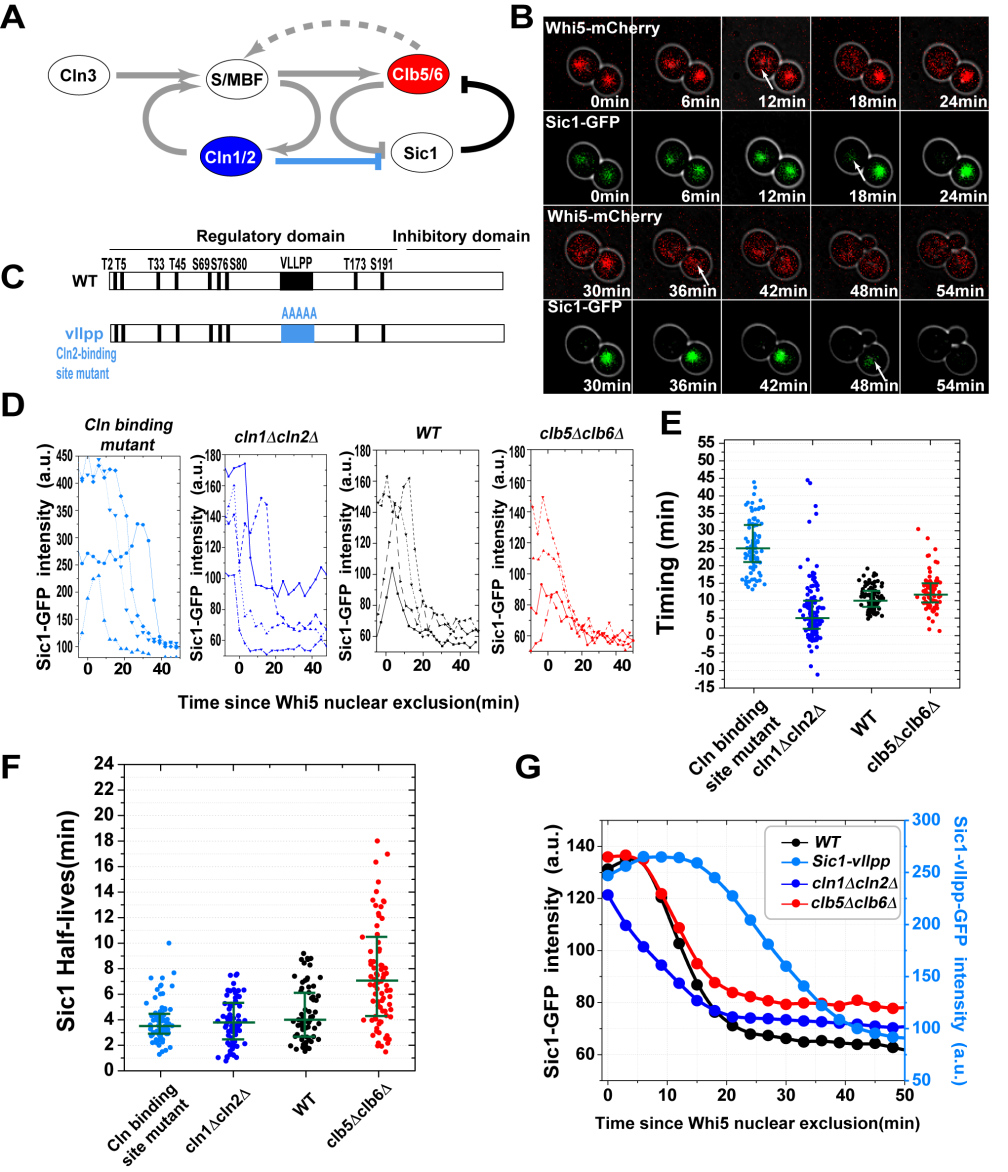
Cln1/2-Cdk1 sets the proper timing of Sic1 destruction. (A) The topology of the G1/S switch. Colored nodes and link are perturbed to assess their role in the timing of Sic1 destruction, as shown in the following panels. (B) Combined phase and fluorescence time-course images in wild-type cells (red, the endogenous Whi5-mCherry; green, the endogenous Sic1-GFP). White arrows indicate the time where Whi5 is being excluded out of nucleus and Sic1 is being degraded, in a mother and a daughter cell, respectively. (C) The construction of the Cln2 binding site mutant of Sic1. (D) Typical Sic1 profiles in various strains. The profiles are aligned at the point of Whi5 nuclear exit (t = 0). (E and F) The initiation time of Sic1 degradation (E) and the Sic1 half-life (F) in wild-type, Cln2 binding site Sic1 mutant, *cln1Δcln2Δ*, and *clb5Δclb6Δ* strains. (G) The average of Sic1 profiles from all single cells, aligned at Whi5 nuclear exit.

To investigate the role of Cln1/2-Cdk1 on Sic1 destruction, we further tagged the transcriptional repressor Whi5 with a red fluorescent protein (mCherry FP), which enabled us to measure the time interval between the Start and the S phase entry. The exclusion of Whi5 from the nucleus marks the point of Start transition at which the transcriptional activity of SBF and MBF is turned on [29]. We define “timing” of Sic1 destruction as the time from Whi5 nuclear exclusion to the point of the fastest Sic1 degradation (Figures 1 and 4B, Figure S3B). Proper timing of Sic1 destruction is essential for a proper S phase entry (Figure S4). We found that the timing of Sic1 destruction was not significantly affected in *clb5Δclb6Δ* strains. In contrast, it was much more variable in *cln1Δcln2Δ* strain (Figure 4D and 4E). The median timing was shorter in *cln1Δcln2Δ* strain, presumably because Clb5/6-Cdk1 significantly contributed to Whi5 nuclear exclusion when Cln1/2-Cdk1 was absent (Figure S3C).

Considering that the dynamics of Whi5 nuclear exclusion is highly affected in *cln1Δcln2Δ* strain (due to the absence of the first feedback loop; Figure 4A), rendering our Start reference point unreliable, we further introduced a Sic1 variant with a mutation on the Cln1/2-Cdk1 binding site (Figure 4C). This mutation disables Cln1/2-Cdk1–mediated phosphorylation of Sic1 [18],[32] without deleting *CLN1/2*, and thus the Whi5 dynamics is unaffected. The destruction timing of this Sic1 mutant was delayed and much more variable than the wild-type (Figure 4E), while the speed of its destruction was as fast as the wild-type (Figure 4F). Taken together, these observations imply that while Clb5/6-Cdk1 is critical for Sic1 fast destruction, Cln1/2-Cdk1 is responsible for setting a robust timing of Sic1 destruction.

To demonstrate how population-level studies can sometimes be misleading, we averaged the Sic1 concentration profile over many individual cells (Figure 4G). The plot shows an apparent more significant effect of Cln1/2-Cdk1 than Clb5/6-Cdk1 on Sic1 degradation, which is clearly due to the more variable timing of the Cln1/2 mutants. This result explains the discrepancy between our single-cell experiments and the earlier population-level studies that reported that deleting *CLB5* and *CLB6* does not affect the rate of Sic1 turnover (compare the black and red lines in Figure 4G) [14],[22].

### A Multisite Phosphorylation Scheme Is Not Necessary to Achieve Rapid Sic1 Destruction

**Figure 5:**
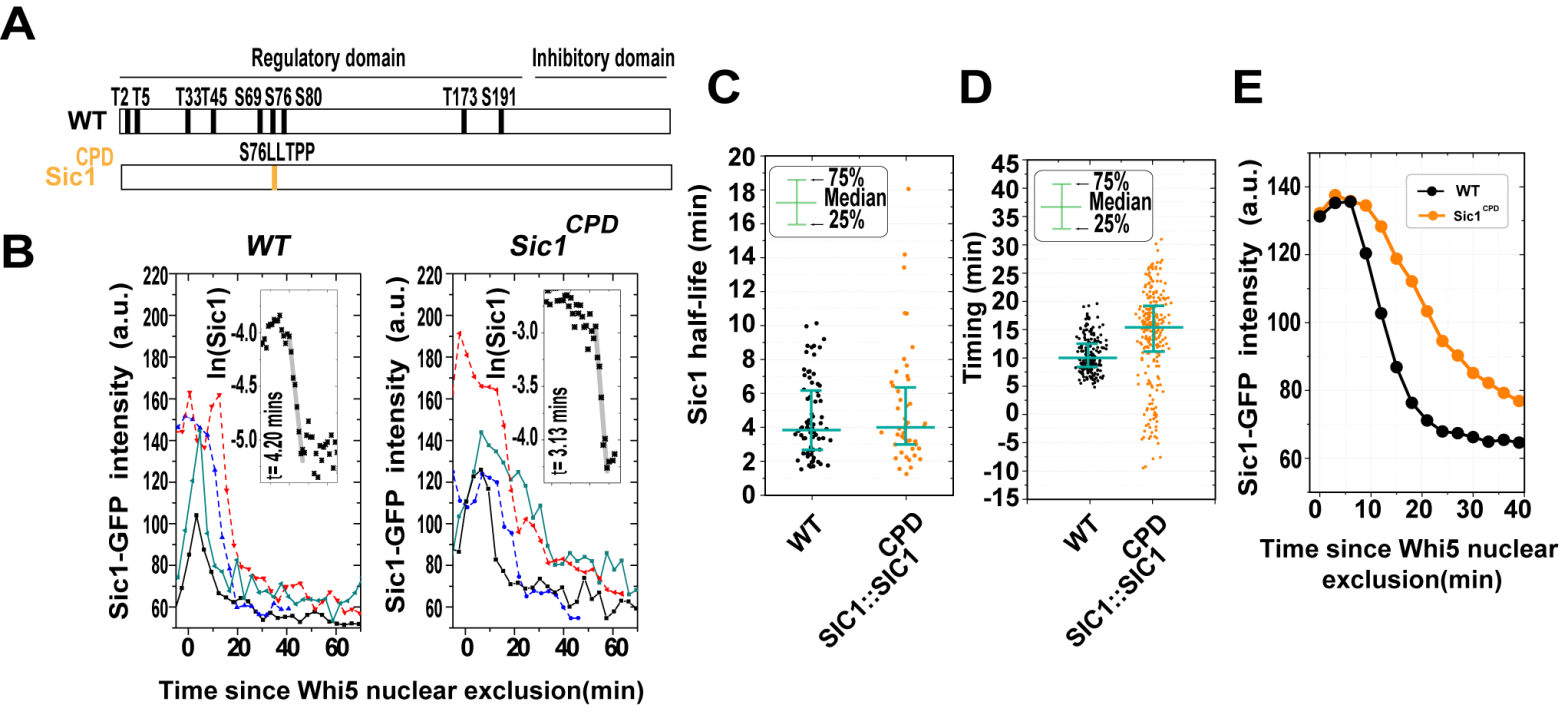
A multisite phosphorylation scheme is not required for fast destruction of Sic1. (A) The construction of the Sic1^CPD^ mutant. (B) Example time course of Sic1-GFP (left) and Sic1^CPD^-GFP (right). Different colors represent different cells. Two mother cells (solid lines) and two daughter cells (dashed lines) are shown. The insets show higher time-resolution (1 frame/min) from a typical cell and fit to exponential decay, along with the fitted half-life. (C) Sic1 half-life and Sic1^CPD^ half-life. Each dot represents a measurement of a single cell. (D) Timing for wild-type and SIC1^CPD^ strains, respectively. Each dot represents a measurement of a single cell. (E) Sic1 and Sic1^CPD^ profile averages over all cells. Single-cell profiles are aligned at t = 0 (Whi5 nuclear exit) for averaging.

An important feature of this circuitry is that Sic1 has to be phosphorylated multiple times before it can be degraded [14],[15],[17]. A study based on Western blotting of Sic1 in synchronized cell populations suggested that the destruction of wild-type Sic1 is more switch-like than a single-phosphosite mutant, Sic1^CPD^ [15]. Sic1^CPD^ lacks all nine endogenous CDK phosphorylation sites of Sic1 but incorporates a single CDK site with a high-affinity Cdc4 binding motif (LLTPP) in place of Ser 76 as shown in Figure 5A [15]. This could imply that the requirement of multisite phosphorylation for Sic1's degradation is responsible for its switch-like destruction [15],[24]. We placed *SIC1^CPD^-GFP* at the chromosomal *SIC1* locus under the control of the endogenous *SIC1* promoter and investigated its destruction dynamics in single cells. We found no obvious difference in half-life between Sic1 and Sic1^CPD^ (Figure 5B and 5C). However, compared to Sic1, a much more variable timing of Sic1^CPD^ destruction was observed (Figure 5D).

Considering that *clb5Δclb6Δ* mutations increase the half-life of Sic1 and that Sic1^CPD^ mutant degrades as fast as wild-type, we conclude that Sic1's fast destruction is not a result of a threshold set for Cln1/2-Cdk1 activity by multisite phosphorylation, suggested by the current model [15].

When we averaged Sic1 and Sic1^CPD^ concentration profiles over many individual cells, we saw that Sic1^CPD^ does appear to degrade slower, consistent with the earlier population-level study [15]. Our result demonstrates that slow Sic1^CPD^ degradation observed in [15] is mainly due to the large cell-to-cell timing variability of Sic1 degradation (Figure 5E).

### Design Principles of Yeast G1/S Switch

**Figure 6:**
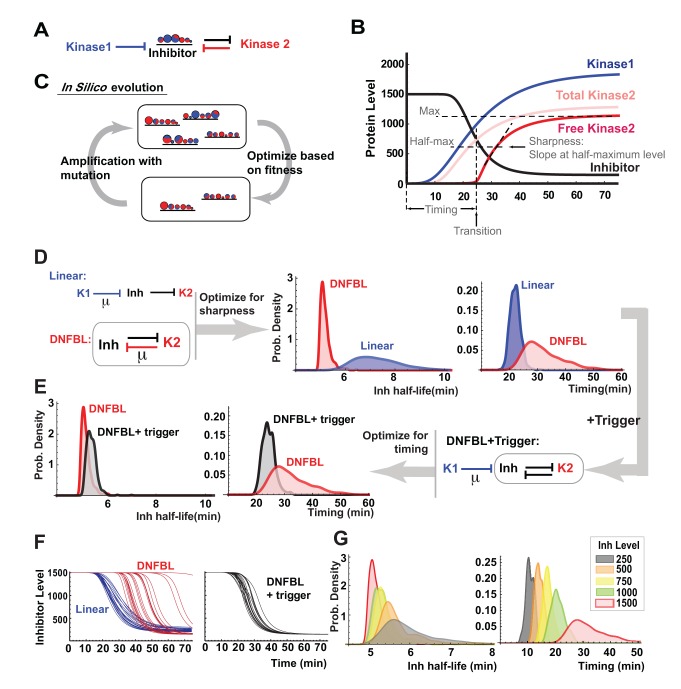
Computational optimization of the circuits for specific functions further reveals general design principles. (A) The mathematical model of the switch illustrated. Small circles on Inhibitor represent phosphorylation sites, and the red and blue shaded areas on each circle represent the magnitudes of catalytic efficiencies of each kinase for that site, schematically. Inhibitor sequesters Kinase2. (B) Definitions of timing and sharpness illustrated on a sample run (see Text S3 for details). (C) *In silico* evolution to optimize the catalytic efficiencies of the kinases for a desired function in discrete steps of mutation and selection (see Figure S5A for a detailed illustration). (D) The double-negative feedback and the linear circuit were optimized for sharpness of Kinase2 activation. Distributions for Inhibitor half-life and activation timing after optimization are shown on the right. (E) An upstream trigger is added to the double-negative feedback circuit that is already optimized for sharpness. The circuit is then optimized for timing by mutating the catalytic efficiency of Kinase1 only. The resulting circuit greatly reduced the timing variability, but also slightly decreased sharpness. (F) Dynamical behavior of each design is shown using the data from (D) and (E). Linear circuit produces consistent timing, but variable sharpness; double-negative feedback produces consistent sharpness, but variable timing. Collaboration of two kinases leads to robustness in both timing and sharpness in Kinase2 activation (see Figure S5C for a more detailed illustration). (G) The trade-off between timing and sharpness shown in a double-negative feedback loop with various levels of Inhibitor.

To further elaborate on the design principles of the G1/S switch and to place our experimental findings on a more general footing, we also carried out a computational study. We constructed an analogous ODE model of the switch consisting of three components (named Kinase1, Kinase2, and Inhibitor) to identify the contributions of the components of the switch to the sharpness and timing of the transition and their respective robustness under cell-to-cell variability on the inputs of the switch (Figures 6A–C) [33]. Essentially, we incorporated extrinsic noise on Kinase1 and Kinase2 production and degradation by varying the synthesis and degradation rates from realization to realization (Text S3). We then optimized the phosphorylation rates to obtain either the sharpest or most timely Kinase2 activation (Figures 6A–C and S5A). We did not incorporate noise in any other reactions and kept the Inhibitor level constant. Note that even though only extrinsic noise was incorporated in this simplified model, the results are applicable to cases where intrinsic noise is also present. (The simplified model is different than the stochastic model of the entire G1/S circuitry, whose results are presented in Figure 2G.)

First, we compared the linear circuit (suggested by earlier studies) and the double-negative feedback to discern the roles of multisite phosphorylation and topology on the sharpness of the Inhibitor destruction. We optimized the catalytic efficiencies of the kinases in both circuits to obtain the sharpest Kinase2 activation, as a measure of the decisiveness of the switch (Figure 6D). The double-negative feedback yielded sharper Inhibitor destruction on the average than the linear circuit, confirming that the topology is the dominant factor in determining sharpness and its robustness (i.e., decisiveness), and not multisite phosphorylation.

Double-negative feedback is more effective in generating sharp Inhibitor destruction [34] because in the linear topology case, speed of degradation is directly affected by the variability in Kinase1 level. The feedback loop ensures that the Inhibitor degradation does not begin before sufficient amount of Kinase2 is accumulated; therefore, it buffers the variability. The disadvantage of the double-negative feedback circuit is that the timing is highly variable for the same reason (last panel in Figure 6D and 6F). The activation occurs when Kinase2 level exceeds the Inhibitor level. The time of the activation varies highly, because Kinase2 approaches to its steady-state level asymptotically, and we assume that the initial level of the Inhibitor and the steady-state level of Kinase2 are comparable, and Kinase2 approaches to that level asymptotically as illustrated in Figure S5D. (The assumption is due to the fact that lower levels of Inhibitor yield timing variabilities that do not match the timing variability of Cln1/2 deletion, as seen in right panel of Figure 6G and Figure S5B.) This is a trade-off that underlies the design of the switch: increasing the Inhibitor level increases the sharpness of the Kinase2 activation and Inhibitor degradation; however, it also increases the variability of the timing (Figure S5D and Figure 6G).

An interesting fact about the linear circuit is that it generates robust timing, even though it was optimized for sharpness. We next used this insight to see if timing variability of the double-negative feedback loop circuit could be improved by adding an upstream trigger. We picked a double-negative feedback circuit from the sharpness-optimized set (Figure 6D) and attached Kinase1 to it. We then optimized this circuit for timing by only mutating the Kinase1 phosphorylation rates on Inhibitor. Indeed, addition of the trigger reduced the standard deviation of the timing distribution by more than 65% (Figure 6E). This is because Kinase1 enforces timely activation of Kinase2 by initiating Inhibitor degradation (Figure 6F). However, the trade-off is seen here as well—this modification reduced the sharpness of the transition and increased its variability, since Kinase2 activations occurred at a lower level of the Inhibitor on the average. This result is consistent with the Cln2-Cdk1 binding-site mutant of Sic1 that yielded slightly sharper transitions with a lower variability compared to wild-type (Figure 4F). The model predictions qualitatively agree with the *clb5Δclb6Δ*and Cln2-Cdk1 binding-site mutant experiments (Figure S5B).

In light of the mathematical model, it is clear that Sic1 plays two roles in the G1/S switch by setting a threshold for Clb5/6-Cdk1 activity. First, it prevents precocious activation of Clb5/6-Cdk1. Second, it allows accumulation of a large stockpile of Clb5/6-Cdk1 prior to activation. This stockpile is responsible for the consistently fast (i.e., switch-like) destruction of Sic1, leading to a sharp rise in Clb5/6-Cdk1 activity, buffering the variability in Clb5/6 level. It is possible to drive the transition only by Clb5/6-Cdk1 [35]. In this case, Sic1 degradation begins when Clb5/6-Cdk1 exceeds the Sic1 level, given that Sic1 inhibits Clb5/6-Cdk1 tightly [34]. Our model shows that this event will occur with a large timing variability if the rate of increase in Clb5/6 slows down as it approaches to Sic1 level. Cln1/2-Cdk1 corrects the timing of the double-negative feedback loop by initiating Sic1 degradation to compensate for the asymptotic approach of Clb5/6 to its steady-state level. It seems like timing variability can also be reduced by increasing the steady-state level of Clb5/6-Cdk1. Why nature chose to use Cln1/2-Cdk1 instead is an open question.

## Discussion

### A Clear Picture of S Phase Entry

Given its essential role in guarding genome integrity, Sic1 destruction has been studied extensively within the last two decades. However, a clear understanding of the dynamic nature of the S phase entry did not emerge due to the discrepancies between the studies and lack of single-cell experiments.

**Figure 7:**
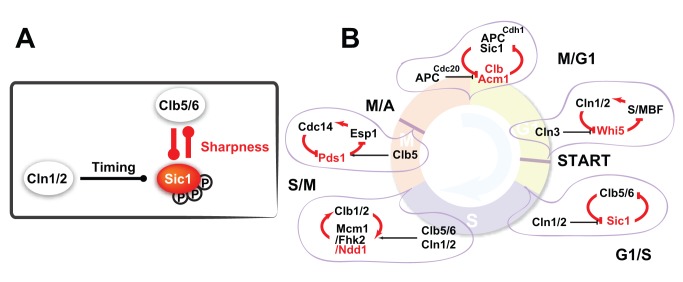
A common motif of biochemical switch. (A) The new model of the G1/S transition. (B) The switch motif at the various transitions in cell-cycle regulation.

In this article, we monitored the dynamics of Sic1 destruction in real time and in single cells, and provided a dynamic picture of the G1/S transition in yeast.. Our experiments show that both Cln1/2-Cdk1 and Clb5/6-Cdk1 contribute to Sic1 destruction *in vivo*. The role of Cln1/2-Cdk1 is to set the proper timing of Sic1 destruction, whereas the double negative feedback loop between Sic1 and Clb5/6-Cdk1 ensures the robustness of the destruction speed. The double-negative feedback loop functions as a noise filter, and is essential for the robust S-phase entry under genetic and environmental perturbations (Figure 7A). Interaction between Sic1 and Clb5/6-Cdk1 is analogous to the interaction between Rum1 and Cdc13-Cdc2 studied in [36],[37].

At first glance, involvement of two kinases in the phosphorylation of Sic1 seems like a redundancy. Sic1-Clb5/6-Cdk1 double-negative feedback loop generates irreversible and robustly sharp Sic1 destruction [38], and Clb5/6-Cdk1 is capable of triggering its own activation [34],[35]. Timing of the transition (i.e., when Clb5/6-Cdk1 level exceeds the Sic1 level) can be tuned by adjusting the synthesis/degradation rates of Clb5/6. Then why does Cln1/2-Cdk1 initiate the Sic1 degradation? A possible reason could be that Cln1/2-Cdk1 transcription initiation serves as a reference for the Start transition, which can be used to time the subsequent cell-cycle events. However, it is hard to imagine why Clb5/6 cannot serve as a reference since Cln1/2 and Clb5/6 are both on the G1/S regulon—that is, their transcription is coupled [39]. Our model suggests that Clb5/6-Cdk1 is not used to time its own activation because its asymptotic approach to steady state makes timing unreliable (second panel in Figure 6F and Figure S5D). Timing variability is reduced dramatically when Sic1 destruction is initiated by Cln1/2-Cdk1 as Clb5/6 rises. Collaboration of two kinases brings robustness in timing and speed of destruction of Sic1.

Sharing substrates between CDKs is almost a signature of the cell cycle control system. Here in this case, we provide an important functional implication for this apparent redundancy. Indeed, the regulatory motif we study here, a trigger coupled to a double-negative (or positive) feedback loop, occurs in the regulation of virtually every cell cycle transition (Figure 7B) [38].

### Function of Multisite Phosphorylation

Multisite phosphorylation of Sic1 by Cln1/2-Cdk1 was thought to be responsible for the switch-like destruction of Sic1 at the S phase entry [15],[24]. In principle, multisite phosphorylation can set a threshold for CDK activity and generate an ultrasensitive response. However, our experiments show that neither Cln1/2-Cdk1 nor a multisite phosphorylation scheme is necessary for switch-like destruction of Sic1. First, it is Clb5/6-Cdk1-mediated phosphorylation (but not Cln1/2-Cdk1) that leads to a rapid destruction of Sic1. Second, the threshold for Clb5/6-Cdk1 activity is set by Sic1 inhibition, not by multisite phosphorylation. Third, a single, optimized phosphosite on Sic1 can generate just as a rapid destruction as the wild-type. Our results are consistent with the recent theoretical studies, which suggest that while multisite phosphorylation can effectively establish a threshold, the response beyond the threshold may not be switch-like [25], and a sharp transition that relies on one-step “linear” ultrasensitivity alone may be prone to cellular noise [40].

We emphasize that, on wild-type Sic1, phosphorylation of multiple sites is required for a rapid destruction (see the docking network shown in figure 3 in [18]). In other words, no single site on wild-type Sic1 has a high enough affinity for Cdc4 to promote a rapid destruction. The mutant we use, Sic1^CPD^, has an optimized phosphosite specifically for this purpose. The result that Sic1^CPD^ degrades as fast as the wild-type does not suggest that multisite phosphorylation is redundant. It is likely that multisite phosphorylation of Sic1 integrates Cln1/2-Cdk1 and Clb5/6-Cdk1 signals to optimize the response of the switch to its inputs. Besides the possibility of helping with the switching dynamics, it has been reported that some phosphorylation sites are important for cells to respond to stress [41]. This suggests that a benefit of having multiple phosphorylation sites is to interpret signals from other signaling pathways.

The requirement for multisite phosphorylation could potentially allow Sic1 to ignore low levels of Cln-CDK activity in early G1 phase and thus, in principle, could also help with the timing. Indeed, it seems the multisite phosphorylation does help with the timing in view of the Sic1^CPD^ case. However, given the fact that the phosphorylation site of Sic1^CPD^ is located on S76, which is a very Clb5-Cdk1 favored site [18], we cannot rule out the possibility that the large timing variability observed there is simply due to the lack of Cln1/2-Cdk specificity; and a single phosphorylation site may be able to achieve robust timing with some tuning.

### Population versus Single-cell measurements

Most previous *in vivo* studies of Sic1 destruction dynamics have mainly relied on Western blotting of synchronized yeast populations, which, due to large cell-to-cell variability and intrinsic noise, do not accurately report the actual Sic1 concentration as a function of time in individual cells. As demonstrated in this work and some other works as well [29], some dynamic features of a system are easily discernible at the level of individual cells but may be hard to detect at the population level, and population average of protein dynamics sometimes can lead to erroneous conclusions.

### The Contribution of Other B-Type Cyclins and Cdh1

Besides Clb5 and Clb6, there are four other B-type cyclins in budding yeast. They all can phosphorylate Sic1 efficiently *in vitro*. Clb1–4 do not contribute to the DNA replication onset in wild-type, suggesting that they do not play a role in Sic1 destruction under normal conditions. However, since Clb1–4 initiates DNA replication in the absence of Clb5 and Clb6, we cannot rule out the possibility that Clb1–4 contribute to the Sic1 destruction in absence of Clb5 and Clb6. If that is the case, the Sic1 destruction with Cln1/2-Cdk1 activity only could be even slower than we observed in *clb5Δclb6Δ* cells.

Some previous experiments suggested that Cdh1 is phosphorylated by Clb5 *in vivo* [42], which could imply that Cdh1 may be capable of forming another double negative feedback loop with Clb5/6 at G1/S transition. Therefore, we also investigated the potential function of Cdh1 in Sic1 destruction dynamics. We first monitored the Sic1* half life in *cdh1Δ*, and we found that it is similar to wild-type (Figure S5). Then we further perturbed the system by either deleting *CLN1* and *CLN2* in the *cdh1Δ* strain, or subjecting the *cdh1Δ* cells to stress (1 M KCl). In all cases, Sic1* half-life is similar to wild-type (Figure S2D, E), suggesting that Cdh1 does not contribute significantly to Sic1destruction dynamics.

In conclusion, our study reveals the design principles of the biochemical switch at the G1/S transition: (1) the feedback loop between Clb5/6-Cdk1 and Sic1 is responsible for generating a sharp Clb5/6-Cdk1 activation robustly, and (2) Cln1/2-Cdk1 initiates Sic1 degradation and sets the timing of the activation (Figure 6F and Figure S5C). It is known that cyclin-CDKs often share their substrates in cell-cycle regulation. In this case we see that the apparent “redundancy” of Sic1 being a substrate of both Cln1/2-Cdk1 and Clb5/6-Cdk1 is an important part of the design of the G1/S switch. The regulatory motif we study here, a trigger coupled to a double-negative (or positive) feedback loop activated through multisite phosphorylation, occurs in the regulation of virtually every cell cycle transition (Figure 7B) as well as in other pathways [43], highlighting its utility in achieving both robust timing and decisive switching.

## Methods

### Yeast Strains

Standard methods were used throughout. Most strains used in this study were congenic S288c, except the *SIC1-0P* strain from Frederick R. Cross [44]. All *SIC1* mutants were introduced at the chromosomal *SIC1* locus under the control of the endogenous *SIC1* promoter. The *KanMX/NAT/LEU2* fragments, flanking with homologous sequence to the target gene (∼40–50 bp), were used to delete genes. Genotypes of deletion strains were tested by PCR. All single mutant strains were characterized by sequencing PCR products.

The plasmid pCT03 (*pGREG506-ADH1pr-mCherry*) was obtained by subcloning *ADH1* promoter (starting from 713 bp upstream of the start codon of gene *ADH1*, and PCR from the genome) into the *SacI-NotI* site and inserting the *NotI-SalI* fragment containing mCherry with a 11 amino acid linker (Ala-Ala-Ala-Gly-Asp-Gly-Ala-Gly-Leu-Ile-Asn-) [45] at the N terminal (PCR from pNT11, a kind gift of Jonathan Weissman (UCSF)). Plasmids pCT04, pCT05, and pCT06 were constructed by inserting *NotI-NotI* fragments containing the regulatory domain of Sic1 (1–220 aa, PCR from the genome), MCM marker (PCR from pML103, a kind gift of Joachim Li (UCSF) [46]), and *HTB2* (PCR from the genome), respectively. The plasmid pCT07 was constructed by inserting GFP (PCR from the plasmid pNT10, a kind gift of Jonathan Weissman (UCSF)) at *SpeI-SalI*, and inserting the *PEST* sequence of *CLN2* [47] (PCR from the genome) at *SalI* of the plasmid pGREG533. Then plasmids pCT08, pCT09, and pCT10 were constructed by inserting *CLN2* promoter (starting from 656 bp upstream of the start codon of the gene *CLN2*, PCR from the genome), *CLB5* promoter (starting from 800 bp upstream of the start codon of the gene *CLB5*, PCR from the genome), and *SWI4* promoter (starting from 1,068 bp upstream of the start codon of the gene *SWI4*, PCR from the genome). All plasmids were characterized by sequencing.

### Time-Lapse Microscopy

Cells growing exponentially in synthetic liquid medium were seeded onto thin 1.5%–2% agrose slabs of the same medium. Multiple different positions were followed simultaneously. Images were acquired every 1 min for Sic1 half-life experiments, 3 min for timing experiments, and 7 min for promoter experiments.

For endogenous Sic1 experiments, fluorescence and phase microscopy were performed using a TE2000E Inverted Microscope (Nikon) with perfect Focus, Apo 60×/1.40 NA oil-immersion objective, a Cascade II 512 CCD (Photometrics), and NIS-Elements Advanced Research software. The microscopy experiments of Sic1 reporter and promoters were performed in the University of California San Francisco Nikon Imaging Center using a TE2000E Inverted Microscope (Nikon) with perfect Focus, Apo 60×/1.20 NA water-immersion objective, a Coolsnap HQ2 Camera (Photometrics), and NIS-Elements Advanced Research software (http://nic.ucsf.edu/timelapse.html). Potential toxicity of fluorescence illumination was tested with pre-experiments.

### Image Analyses

Image segmentation and fluorescence quantification were performed using custom Matlab software and ImageJ. Custom Matlab software was used to fit an exponential function to the fluorescence data to obtain the half-life of the endogenous and reporter Sic1 in various strains.

### Half-Life Analysis

Fluorescence signals from endogenous Sic1 or Sic1 reporter/mutants were extracted from the images. We fitted an exponential function to the signal intensity data to obtain the half-life of the protein (Figure 2C, Text S1).

For Sic1 reporter, we fitted the function directly onto the mean reporter fluorescence signal from the maximum-intensity 5×5 square. For endogenous Sic1, we first divided the mean nuclear Sic1 intensity by the mean nuclear label intensity and fitted the function onto the resulting values.

The function we fitted to the fluorescence signal is
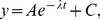
(1)where *y* is the time series of fluorescence signal. However, since this function will cause a bias towards larger values, we also fit the signal to this form of the function:
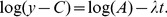
(2)


To fit using Eq. 2, we first found the constant *C* that gives the straightest line in the log domain when subtracted from the data. Then we fitted a straight line to the log subtracted data and the absolute value of the slope is the degradation rate.

### Stochastic Model

The chemical Langevin equation [48] was used to simulate the intrinsic noise. Extrinsic noise was simulated by randomizing the model parameters around their nominal values within a certain percentage range. Sic1 half-life was measured in many realizations of the stochastic simulation. See Text S2 for details.

### *In Silico* Evolution

All simulations start with low concentrations of kinases and a high concentration of Inhibitor. Kinase1 and Kinase2 have identical synthesis and degradation rates, but their profiles vary as the rates are subject to extrinsic noise (Figures 6B,F and S5C). Inhibitor level does not vary. We define timing of the transition as the time when Kinase2 level exceeds that of Inhibitor, and sharpness as the slope of free Kinase2 at its half-maximum level.

We start the simulation with 1,000 copies of the switch, each assigned a random set of Kinase1 or Kinase2 catalytic efficiencies (i.e., phosphorylation rates) at each phosphosite. We run each copy and calculate a fitness score based on timing or sharpness of the transition. We eliminate half of the population with lowest scores and duplicate the survivors to make up the deficit. Lastly, we mutate the catalytic efficiencies for each phosphosite in each duplicate by a small rate. We repeat this process for 1,000 generations. We assume that phosphorylation is ordered—that is, site 1 is phosphorylated first, site 2 is phosphorylated second, etc. See Text S3 for details.

## Supporting Information

Figure S1
**GFP-fused Sic1 has a similar half-life to that of endogenous Sic1 (A–C) and Sic1 half-life measurements in single cells (D–I) (supplement for Figure 2).** (A) Cells, containing either *GAL1pr-SIC1-HA* or *GAL1pr-SIC1-GFP*, were arrested by alpha factor for 1.5 h, and then GAL promoters were turned on for 30 min. Cells were released from alpha-factor arrest after 2.5 h, and immunoblotted for total Sic1 protein. (B) Half-lives of Sic1-HA and Sic1-GFP were obtained by fitting Western blot data to an exponential decay function, and the error bars were from three independent experiments. (C) There was a linear relationship between Sic1 concentration and signal intensity. Sic1-GFP (left), Sic1-HA (right). Serial dilutions of samples at time point 0 were used to assess the linear dynamic range of the Western blot. The same loading as the last lane here was used for the half-life measurement (time point 0) in (A). (D) Images from time-lapsed fluorescence microscopy were analyzed using custom software written in Matlab. (Left) Cell segmentation is done automatically from the time series of bright field images, and the software also allows manual correction for the segmented cells (a blue polygon is used to interact with user). (Right) Nuclei are segmented from the fluorescently labeled nuclear images (segmented nuclei are enclosed by yellow lines). (E) The median in the measured Sic1 half-life is slightly increased in the 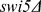 strain, suggesting that although the down-regulation of Swi5 at G1/S may slightly contribute to the observed decrease in Sic1 concentration, the major effect in Sic1 reduction is from its phosphorylation by Cln-Cdk1 and Clb-Cdk1. (F) Half-life of endogenous Sic1 in different deletion strains (mother versus daughter cells). Each dot represents the half-life from a measurement of a single cell. (G) Stochastic simulation results for Sic1 half-life (intrinsic noise only). (H) No significant correlation was found between Sic1 half-life and the time for division to bud in 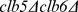 mother cells. Each dot represents a single cell. (I) No significant correlation was found between Sic1 half-life and the time for bud to re-division in 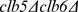 cells. Each dot represents a single cell.(TIF)

Figure S2
Sic1* half-life (A–B) and promoter activity(C) measurement in single cells, and the contribution of Cdh1 (D–E) (supplement for Figure 3). (A) Half-life of Sic1* in various deletion strains. Each circle represents the half-life from a measurement of a single cell. Their behaviors are very similar to Sic1 half-life shown in Figure 2 of the main text. (B) Sic1* does not inhibit Clb5/6-Cdk. The panel shows the comparison of the initiation of DNA replication between wild-type cells and Sic1* overexpressing cells. Cells were first arrested with alpha factor for 2 h, and then GAL1 promoter driven Sic1* was induced by addition of galactose for 2 h; DNA replication was monitored by FACS. (C) Growth rate of WT and sic1 cells under different stress. (D) Sic1* half-life in 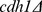 under genetic perturbations. (E) Sic1* half-life in 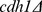 under environmental perturbations. (F) The DNFBL was perturbed by deleting *SIC1*. The cells were further subjected to genetic perturbations as indicated below the data points (black, mother cells; red, daughter cells). (G) The DNFBL was perturbed by deleting *SIC1*. The cells were further subjected to environmental perturbations as indicated below the data points (black, mother cells; red, daughter cells). (H) The DNFBL was perturbed by deleting the link between Clb5/6-Cdk1 and Sic1, which was accomplished by using the nonphosphorylatable Sic1-0p. The cells were further subjected to environmental perturbations as indicated below the data points (black, mother cells; red, daughter cells).(TIF)

Figure S3
**Timing measurement in single cells (supplement for Figure 4).** (A) The fluorescence time-course images of marker MCM (red) and the *CLN2pr-GFP-PEST* (green) in WT. (B) Example time course of Sic1-GFP and Whi5-mCherry used to extract the timing. (Upper panel) Raw data with smoothing spline fitting. (Lower panel) First derivative of the spline curves in the upper panel. (C) Timing of Sic1 destruction in various strains. (D) Timing CV in various strains. (E) Clb5/6-Cdk contributes to the first feedback loop when Cln1/2-Cdk is absent. *CLB5* promoter activation after *SIC1* deletion, compared with wild-type (WT) (upper) and in 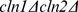 (lower). Right panels are experimental data from individual cells. Each thin curve represents the promoter activity in a single cell, while thick curve represents the mean. Black and red represent cells with and without *SIC1*, respectively. Cells are aligned with the time-point when the promoter turns on (t = 5), and in order to compare the strength of the feedback loop, profiles are normalized to 1. Left panels are corresponding network.(TIF)

Figure S4 **Altered Sic1 destruction dynamics causes genome instability (supplement for Figure 5).** (A) The construction of the Sic1 mutants used in this study. Nine single mutants were constructed by mutating each and every CDK phosphorylation site. The mutant Sic1^CPD^ is constructed by replacing 5 amino acids centered on S76 with the sequence indicated. All other eight CDK sites are mutated to either A or V. The Sic1^CPD^ used in Nash et al. [15]. Due to technical difficulties, the Sic1^CPD^ used in their study had two phosphorylation sites (T2, T5) un-mutated (“Yeast strains and culture” part). (B) Combined phase and fluorescence time-course images in different Sic1 mutants. The SIC1 mutant is tagged with GFP and placed on the endogenous promoter. Note that for the SIC1-45A mutant green fluorescent signal can be seen in the whole population, suggesting that the cell fails to degrade Sic1-45A in one cell cycle. (C) Experimental data of Sic1* half-life in WT and SIC1^CPD^ strain; each circle represents a measurement from a single cell. Note that Sic1^CPD^ has similar half-life as Sic1 even under stressed conditions. (D) Experimental data for the half-life of various mutants. Different phosphorylation sites contribute differently to Sic1 half-life. Each point represents the half-life from a measurement of a single cell. (E) Experimental data of the timing of Sic1 degradation initiation for various mutants. Each point represents a measurement from a single cell. (F) Many Sic1 mutants exhibited an elevated mitotic loss compared with the wild type. See Methods for details about measuring genome instability. Error bars were from four independent measurements.
(TIF)

Figure S5
**The mathematical model (supplement for Figure 6).** (A) *In silico* evolution algorithm illustrated. Simulation begins with *N* copies of the switch each assigned a random set of phosphorylation rates for the phosphosites on Inhibitor (Kinase1 and Kinase2 are not shown). Each copy of the switch is run under extrinsic noise—that is, synthesis and degradation rates of kinases vary at each run. Inhibitor is not subject to noise. After each run, a fitness score is calculated based on a desired characteristic (in this case, timing of the transition). Half of the population with higher fitness is kept; the rest are eliminated. Survivors are duplicated to make up the deficit. Duplicates are mutated–that is, phosphorylation rates for each phosphosite and each kinase are modified with a small probability. Steps 2–4 are repeated for many generations. (B) Comparison between the simulations and experiments. Each curve in the model results was averaged over five runs. For timing, simulations predict similar distributions for WT and 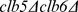, and a larger mean and variability for the Cln1/2-Cdk1 binding mutant. Slightly larger variability seen in 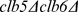 cells compared to the WT. This is most likely due to slower Whi5 phosphorylation since Clb5/6-Cdk1 is not present (absolute values do not match because the model uses t = 0 as the time of Start transition). For the half-lives, the model predicts a slightly sharper and less variable distribution for the Cln1/2-Cdk1 binding mutant compared to WT, and a larger mean and variability for 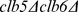. Model distributions are less variable than experimental ones because the simulations are based on ODEs, and Inhibitor level was kept constant for simplicity. Adding noise to the Inhibitor level increases the variabilities about 2-fold (not shown). Distributions from different runs are similar, and do not change the results qualitatively. (C) Dynamics of the designs in Figure 6D–F, shown in detail using the same datasets. (D) Dependence of timing variability in the double-negative feedback loop on Inhibitor level illustrated. Two samples of Kinase2 profiles are shown in the figure. Variability in the profiles is due to extrinsic noise. Kinase2 activation occurs when Kinase2 level exceeds the Inhibitor level. Variability in the timing of activation is lower when Inhibitor level is lower (dark blue arrows). Increasing the Inhibitor level increases the timing variability (light blue arrows) because Kinase2 accumulation slows down as it approaches its steady-state level.(EPS)

Table S1 **Sic1 half-life in different deletion strains.**
(DOC)

Table S2 **Statistical tests of Sic1 half-life distribution.**
(DOC)

Table S3 Sic1* half-life in different deletion strains.
(DOC)

Table S4 Statistical tests of Sic1* half-life distribution.
(DOC)

Table S5 Statistical tests of Sic1* half-life distribution under genetic perturbations (with and without DNFBL).
(DOC)

Table S6 Sic1* half-life under environmental perturbations (with and without DNFBL).
(DOC)

Table S7 Statistical tests of Sic1* half-life distribution under environmental perturbations (with and without DNFBL).
(DOC)

Table S8 **Dynamics of promoters CLN2pr/CLB5pr in different strains.**
(DOC)

Text S1 **Extended experimental procedures.**
(PDF)

Text S2 **Stochastic simulation.**
(PDF)

Text S3 ***In silico*** **evolution.**
(PDF)

## Abbreviations

CDK: cyclin-dependent kinases
DNFBL: double-negative feedback loop
MBF: MluI cell-cycle box binding factor
SBF: Swi4/6 cell-cycle box binding factor
SCF: Skp, Cullin, F-box containing complex
WT: wild-type
