# Supplementary figures and images for: Design Principles of the Yeast G1/S Switch
Source: PLoS Biol. 2013 Oct 1;11(10):e1001673. doi: 10.1371/journal.pbio.1001673 (PMC3794861; doi:10.1371/journal.pbio.1001673)

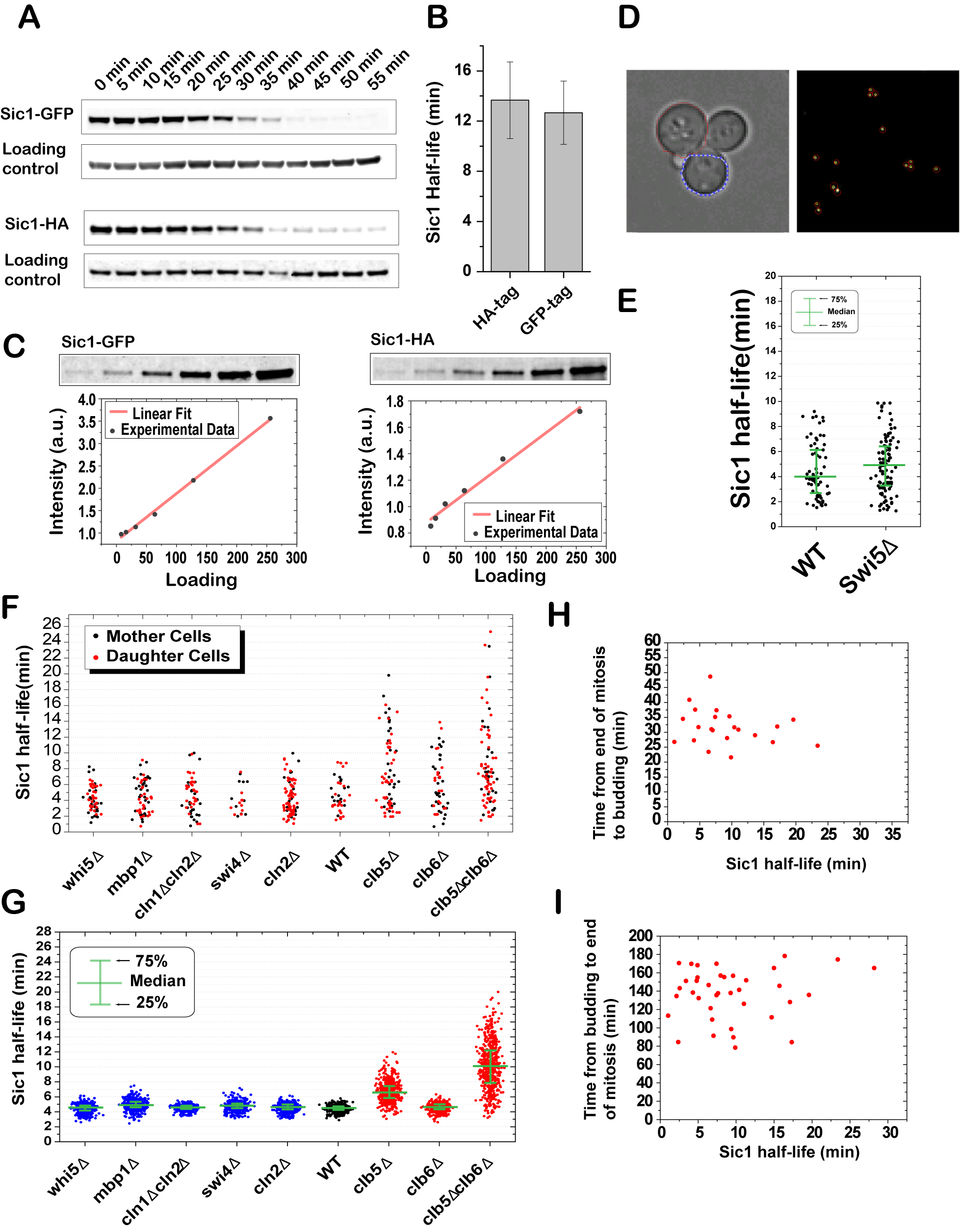

Supplement: Figure S1 — GFP-fused Sic1 has a similar half-life to that of endogenous Sic1 (A–C) and Sic1 half-life measurements in single cells (D–I) (supplement for Figure 2). (A) Cells, containing either GAL1pr-SIC1-HA or GAL1pr-SIC1-GFP, were arrested by alpha factor for 1.5 h, and then GAL promoters were turned on for 30 min. Cells were released from alpha-factor arrest after 2.5 h, and immunoblotted for total Sic1 protein. (B) Half-lives of Sic1-HA and Sic1-GFP were obtained by fitting Western blot data to an exponential decay function, and the error bars were from three independent experiments. (C) There was a linear relationship between Sic1 concentration and signal intensity. Sic1-GFP (left), Sic1-HA (right). Serial dilutions of samples at time point 0 were used to assess the linear dynamic range of the Western blot. The same loading as the last lane here was used for the half-life measurement (time point 0) in (A). (D) Images from time-lapsed fluorescence microscopy were analyzed using custom software written in Matlab. (Left) Cell segmentation is done automatically from the time series of bright field images, and the software also allows manual correction for the segmented cells (a blue polygon is used to interact with user). (Right) Nuclei are segmented from the fluorescently labeled nuclear images (segmented nuclei are enclosed by yellow lines). (E) The median in the measured Sic1 half-life is slightly increased in the strain, suggesting that although the down-regulation of Swi5 at G1/S may slightly contribute to the observed decrease in Sic1 concentration, the major effect in Sic1 reduction is from its phosphorylation by Cln-Cdk1 and Clb-Cdk1. (F) Half-life of endogenous Sic1 in different deletion strains (mother versus daughter cells). Each dot represents the half-life from a measurement of a single cell. (G) Stochastic simulation results for Sic1 half-life (intrinsic noise only). (H) No significant correlation was found between Sic1 half-life and the time for division to bud [file pbio.1001673.s001.tif]

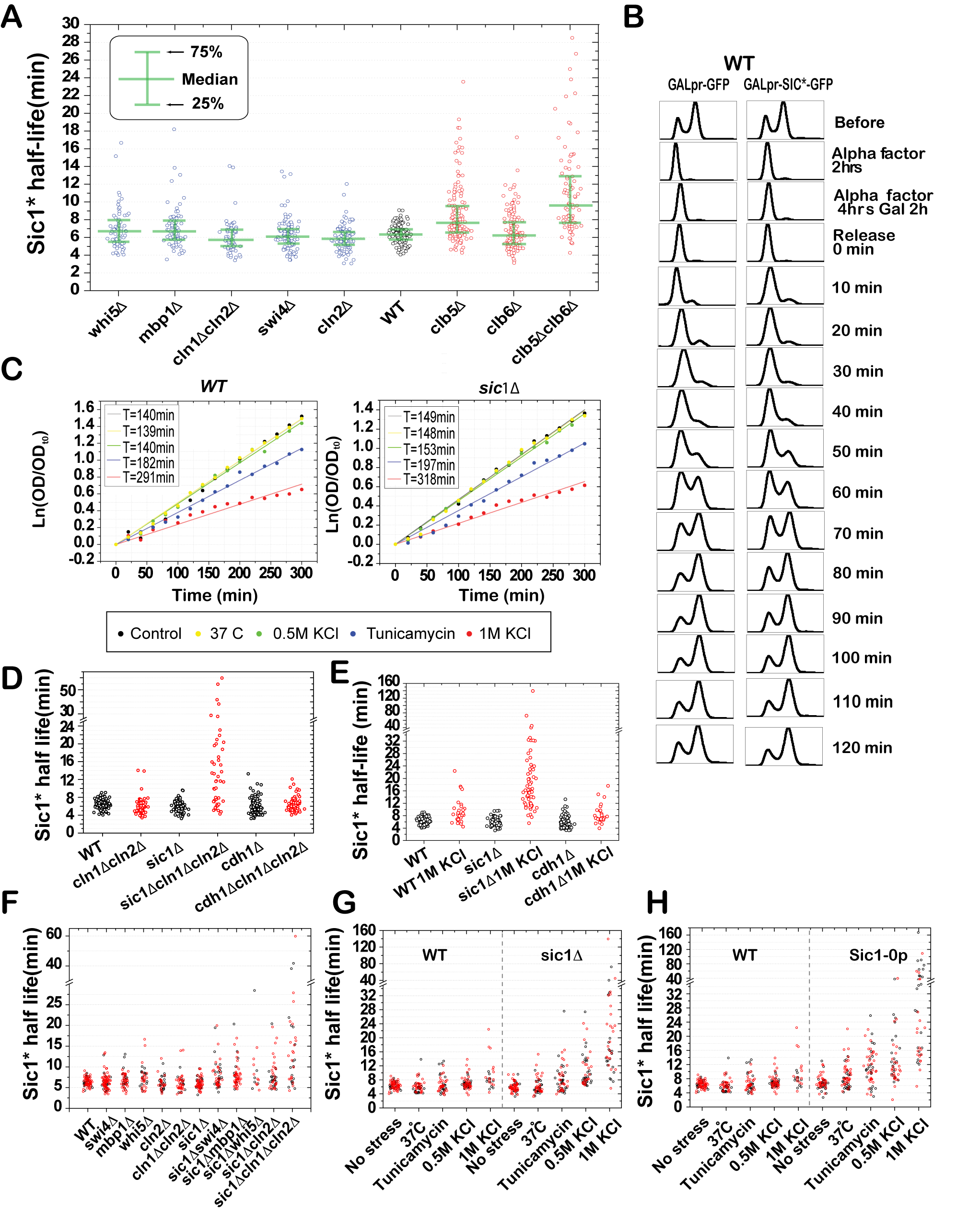

Supplement: Figure S2 — Sic1* half-life (A–B) and promoter activity(C) measurement in single cells, and the contribution of Cdh1 (D–E) (supplement for Figure 3). (A) Half-life of Sic1* in various deletion strains. Each circle represents the half-life from a measurement of a single cell. Their behaviors are very similar to Sic1 half-life shown in Figure 2 of the main text. (B) Sic1* does not inhibit Clb5/6-Cdk. The panel shows the comparison of the initiation of DNA replication between wild-type cells and Sic1* overexpressing cells. Cells were first arrested with alpha factor for 2 h, and then GAL1 promoter driven Sic1* was induced by addition of galactose for 2 h; DNA replication was monitored by FACS. (C) Growth rate of WT and sic1 cells under different stress. (D) Sic1* half-life in under genetic perturbations. (E) Sic1* half-life in under environmental perturbations. (F) The DNFBL was perturbed by deleting SIC1. The cells were further subjected to genetic perturbations as indicated below the data points (black, mother cells; red, daughter cells). (G) The DNFBL was perturbed by deleting SIC1. The cells were further subjected to environmental perturbations as indicated below the data points (black, mother cells; red, daughter cells). (H) The DNFBL was perturbed by deleting the link between Clb5/6-Cdk1 and Sic1, which was accomplished by using the nonphosphorylatable Sic1-0p. The cells were further subjected to environmental perturbations as indicated below the data points (black, mother cells; red, daughter cells). (TIF) [file pbio.1001673.s002.tif]

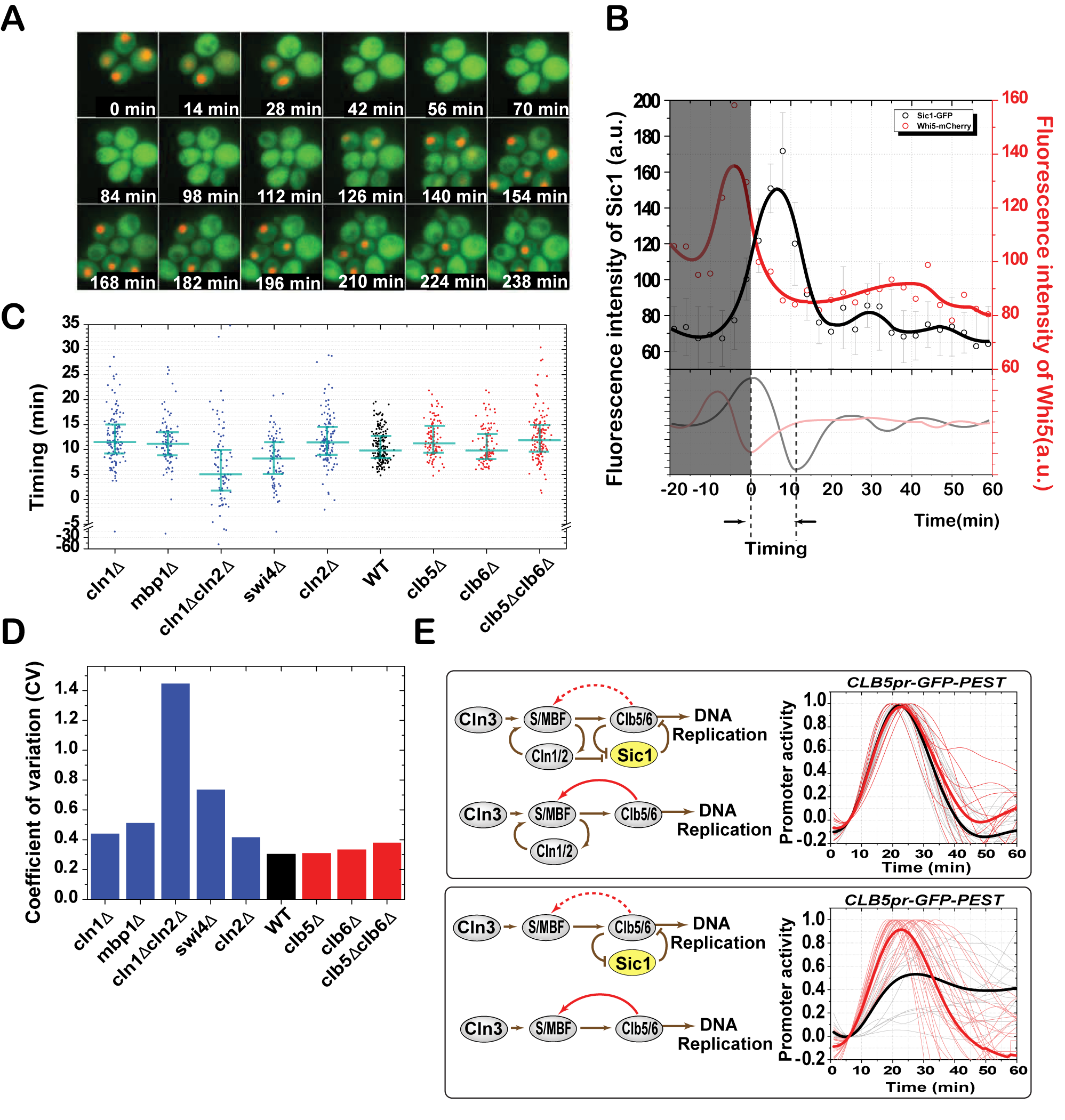

Supplement: Figure S3 — Timing measurement in single cells (supplement for Figure 4). (A) The fluorescence time-course images of marker MCM (red) and the CLN2pr-GFP-PEST (green) in WT. (B) Example time course of Sic1-GFP and Whi5-mCherry used to extract the timing. (Upper panel) Raw data with smoothing spline fitting. (Lower panel) First derivative of the spline curves in the upper panel. (C) Timing of Sic1 destruction in various strains. (D) Timing CV in various strains. (E) Clb5/6-Cdk contributes to the first feedback loop when Cln1/2-Cdk is absent. CLB5 promoter activation after SIC1 deletion, compared with wild-type (WT) (upper) and in (lower). Right panels are experimental data from individual cells. Each thin curve represents the promoter activity in a single cell, while thick curve represents the mean. Black and red represent cells with and without SIC1, respectively. Cells are aligned with the time-point when the promoter turns on (t = 5), and in order to compare the strength of the feedback loop, profiles are normalized to 1. Left panels are corresponding network. (TIF) [file pbio.1001673.s003.tif]

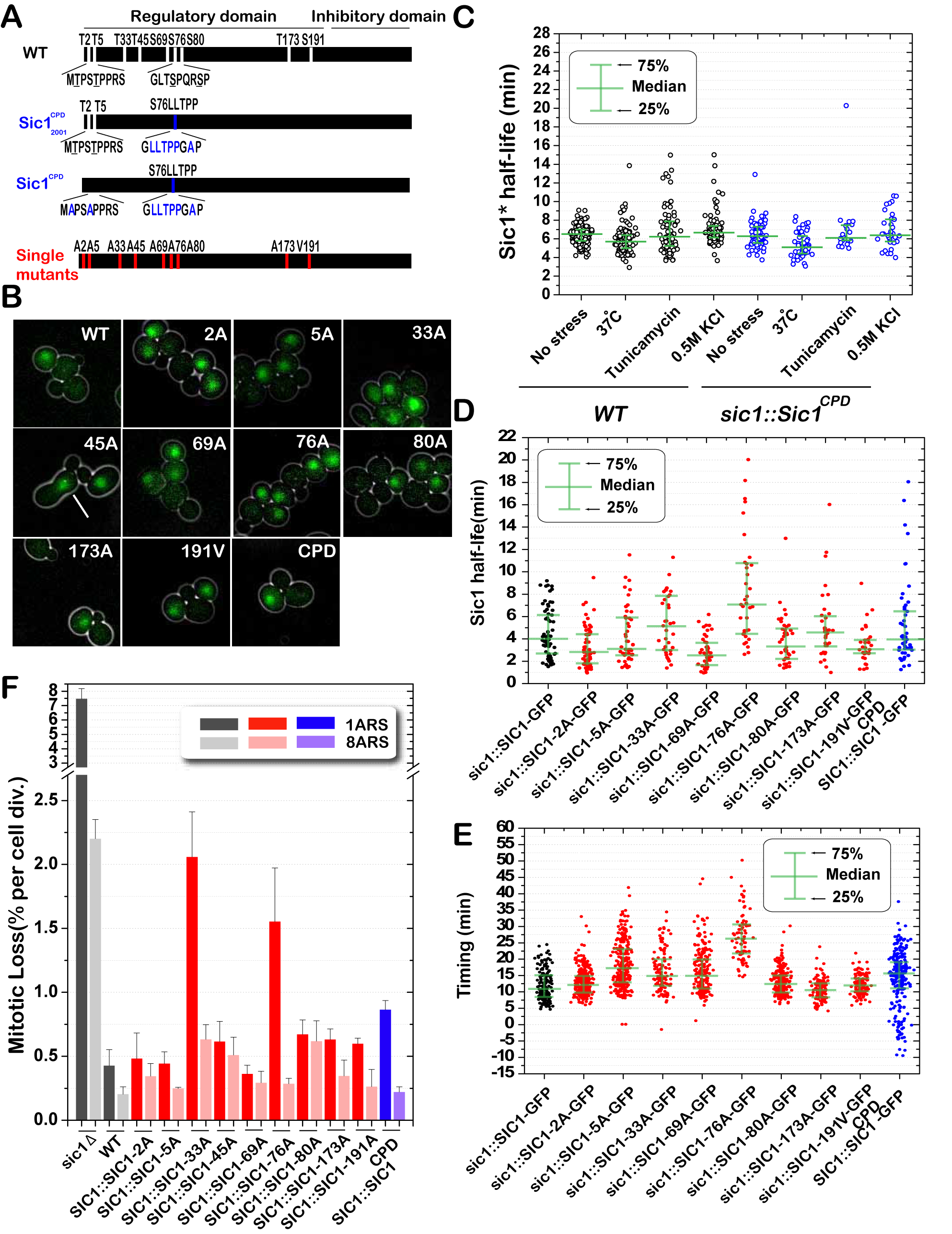

Supplement: Figure S4 — Altered Sic1 destruction dynamics causes genome instability (supplement for Figure 5). (A) The construction of the Sic1 mutants used in this study. Nine single mutants were constructed by mutating each and every CDK phosphorylation site. The mutant Sic1CPD is constructed by replacing 5 amino acids centered on S76 with the sequence indicated. All other eight CDK sites are mutated to either A or V. The Sic1CPD used in Nash et al. [15]. Due to technical difficulties, the Sic1CPD used in their study had two phosphorylation sites (T2, T5) un-mutated (“Yeast strains and culture” part). (B) Combined phase and fluorescence time-course images in different Sic1 mutants. The SIC1 mutant is tagged with GFP and placed on the endogenous promoter. Note that for the SIC1-45A mutant green fluorescent signal can be seen in the whole population, suggesting that the cell fails to degrade Sic1-45A in one cell cycle. (C) Experimental data of Sic1* half-life in WT and SIC1CPD strain; each circle represents a measurement from a single cell. Note that Sic1CPD has similar half-life as Sic1 even under stressed conditions. (D) Experimental data for the half-life of various mutants. Different phosphorylation sites contribute differently to Sic1 half-life. Each point represents the half-life from a measurement of a single cell. (E) Experimental data of the timing of Sic1 degradation initiation for various mutants. Each point represents a measurement from a single cell. (F) Many Sic1 mutants exhibited an elevated mitotic loss compared with the wild type. See Methods for details about measuring genome instability. Error bars were from four independent measurements. (TIF) [file pbio.1001673.s004.tif]
