# Supplementary material for: Design Principles of the Yeast G1/S Switch
Source: PLoS Biol. 2013 Oct 1;11(10):e1001673. doi: 10.1371/journal.pbio.1001673 (PMC3794861; doi:10.1371/journal.pbio.1001673)
Supplement: Table S1 — Sic1 half-life in different deletion strains. (DOC) [file pbio.1001673.s006.doc]

**Table S1. Sic1 half-life in different deletion strains. (Supplement for Figure 2)**

|  |  | **Mother/**  **Daughter percent.** | **Number of Cells** | **Mean**  **(min)** | **s.d.** | **1st quartile** | **Median**  **(min)** | **3rd quartile** |
| --- | --- | --- | --- | --- | --- | --- | --- | --- |
| *whi5Δ* | Total |  | 80 | 4.15 | 1.62 | 2.87 | 4.06 | 5.33 |
|  | Mother | 0.40 | 32 | 3.88 | 1.75 | 2.69 | 3.57 | 4.38 |
|  | Daughter | 0.39 | 31 | 4.43 | 1.31 | 3.31 | 4.27 | 5.78 |
|  | Other | 0.21 | 17 | 4.17 | 1.86 | 2.35 | 4.28 | 5.42 |
| *mbp1Δ* | Total |  | 83 | 4.42 | 2.16 | 2.44 | 4.22 | 6.25 |
|  | Mother | 0.40 | 33 | 4.60 | 2.22 | 2.19 | 4.66 | 6.53 |
|  | Daughter | 0.39 | 32 | 4.10 | 1.96 | 2.50 | 3.91 | 5.63 |
|  | Other | 0.22 | 18 | 4.66 | 2.44 | 2.53 | 3.88 | 6.27 |
| *cln1Δcln2Δ* | Total |  | 78 | 4.20 | 2.16 | 2.58 | 3.89 | 5.71 |
|  | Mother | 0.36 | 28 | 4.16 | 2.49 | 2.23 | 3.53 | 5.56 |
|  | Daughter | 0.37 | 29 | 5.05 | 1.98 | 3.89 | 5.33 | 6.26 |
|  | Other | 0.27 | 21 | 3.07 | 1.30 | 2.01 | 3.41 | 3.90 |
| *swi4Δ* | Total |  | 44 | 4.16 | 2.55 | 2.29 | 3.42 | 5.68 |
|  | Mother | 0.23 | 10 | 4.43 | 2.02 | 2.28 | 4.09 | 6.36 |
|  | Daughter | 0.23 | 10 | 3.78 | 1.58 | 2.71 | 3.52 | 4.03 |
|  | Other | 0.52 | 23 | 3.73 | 2.05 | 2.17 | 3.02 | 5.18 |
| *cln2Δ* | Total |  | 104 | 4.35 | 2.12 | 2.74 | 3.88 | 5.80 |
|  | Mother | 0.24 | 25 | 4.69 | 2.37 | 2.82 | 4.16 | 6.71 |
|  | Daughter | 0.56 | 58 | 4.27 | 2.07 | 2.74 | 3.65 | 5.75 |
|  | Other | 0.19 | 20 | 4.27 | 2.01 | 2.94 | 3.93 | 4.95 |
| *WT* | Total |  | 73 | 4.61 | 2.30 | 2.69 | 3.93 | 6.22 |
|  | Mother | 0.25 | 18 | 4.68 | 1.50 | 3.80 | 4.29 | 5.68 |
|  | Daughter | 0.33 | 24 | 4.33 | 2.37 | 2.54 | 3.48 | 5.95 |
|  | Others | 0.42 | 31 | 4.78 | 2.65 | 2.46 | 3.98 | 7.15 |
| *clb5Δ* | Total |  | 92 | 7.24 | 4.36 | 3.52 | 6.35 | 10.50 |
|  | Mother | 0.32 | 29 | 8.82 | 4.96 | 4.28 | 7.16 | 13.12 |
|  | Daughter | 0.47 | 43 | 6.85 | 4.11 | 3.11 | 6.33 | 10.65 |
|  | Other | 0.22 | 20 | 5.78 | 3.33 | 3.23 | 5.08 | 8.15 |
| *clb6Δ* | Total |  | 80 | 5.95 | 3.31 | 3.00 | 5.08 | 8.28 |
|  | Mother | 0.40 | 32 | 5.86 | 3.33 | 3.25 | 4.89 | 8.71 |
|  | Daughter | 0.26 | 21 | 6.28 | 3.49 | 3.30 | 6.50 | 7.52 |
|  | Other | 0.33 | 26 | 5.54 | 3.01 | 2.72 | 4.72 | 8.33 |
| *clb5Δclb6Δ* | Total |  | 113 | 8.29 | 5.11 | 4.32 | 7.14 | 11.34 |
|  | Mother | 0.24 | 27 | 8.48 | 5.20 | 4.97 | 7.14 | 10.47 |
|  | Daughter | 0.55 | 62 | 8.01 | 5.12 | 4.32 | 6.83 | 9.36 |
|  | Other | 0.21 | 24 | 8.80 | 5.12 | 3.43 | 9.63 | 12.69 |
