# Supplementary material for: Design Principles of the Yeast G1/S Switch
Source: PLoS Biol. 2013 Oct 1;11(10):e1001673. doi: 10.1371/journal.pbio.1001673 (PMC3794861; doi:10.1371/journal.pbio.1001673)
Supplement: Table S2 — Statistical tests of Sic1 half-life distribution. (DOC) [file pbio.1001673.s007.doc]

**Table S2.**  **Statistical tests of Sic1 half-life distribution.** (**Supplement for Figure 2)**

|  | **p values (t- test vs WT)** | **ANOVA+Dunnett's test** | **p values (Mann Whitney test vs WT)** | **Kruskal-Wallis+Dunn test** |
| --- | --- | --- | --- | --- |
| *mbp1Δ* | 0.5932 | 0.9997 | 0.7278 | 0.7073 |
| *cln2Δ* | 0.4412 | 0.9966 | 0.5227 | 0.5549 |
| *swi4Δ* | 0.3308 | 0.9749 | 0.1796 | 0.2273 |
| *cln1Δcln2Δ* | 0.259 | 0.9616 | 0.3587 | 0.3862 |
| *whi5Δ* | 0.1562 | 0.933 | 0.4934 | 0.4719 |
| *clb6Δ* | 0.0045 | 0.0589 | 0.0184 | 0.0218 |
| *clb5Δ** | < 0.0001 | < 0.001 | 0.0002 | 0.0002 |
| *clb5Δclb6Δ** | < 0.0001 | < 0.001 | < 0.0001 | < 0.00001 |
