# Supplementary material for: Design Principles of the Yeast G1/S Switch
Source: PLoS Biol. 2013 Oct 1;11(10):e1001673. doi: 10.1371/journal.pbio.1001673 (PMC3794861; doi:10.1371/journal.pbio.1001673)
Supplement: Table S3 — Sic1* half-life in different deletion strains. (DOC) [file pbio.1001673.s008.doc]

**Table S3. Sic1* half-life in different deletion strains. (Supplement for Figure 3)**

|  |  | **Mother/**  **Daughter Percent.** | **Number of Cells** | **Mean**  **(min)** | **s.d.** | **1st quartile** | **Median**  **(min)** | **3rd quartile** |
| --- | --- | --- | --- | --- | --- | --- | --- | --- |
| *WT* | Total |  | 143 | 6.37 | 0.99 | 5.71 | 6.44 | 6.92 |
|  | Mother | 0.16 | 23 | 6.33 | 0.99 | 5.68 | 6.19 | 6.97 |
|  | Daughter | 0.36 | 52 | 6.43 | 1.02 | 5.74 | 6.42 | 7.18 |
|  | Other | 0.48 | 68 | 6.34 | 0.98 | 5.76 | 6.46 | 6.82 |
| *swi4Δ* | Total |  | 109 | 6.31 | 1.75 | 5.33 | 6.12 | 6.91 |
|  | Mother | 0.17 | 18 | 6.27 | 2.04 | 5.05 | 5.91 | 6.50 |
|  | Daughter | 0.62 | 68 | 6.43 | 1.83 | 5.34 | 6.36 | 7.04 |
|  | Other | 0.21 | 23 | 5.99 | 1.24 | 5.22 | 5.89 | 6.90 |
| *mbp1Δ* | Total |  | 79 | 7.07 | 2.26 | 5.79 | 6.65 | 7.85 |
|  | Mother | 0.24 | 19 | 7.26 | 2.29 | 5.88 | 6.44 | 7.98 |
|  | Daughter | 0.41 | 32 | 6.56 | 1.51 | 5.50 | 6.64 | 7.97 |
|  | Other | 0.35 | 28 | 7.52 | 2.84 | 5.79 | 7.15 | 7.76 |
| *whi5Δ* | Total |  | 65 | 7.04 | 2.34 | 5.50 | 6.71 | 8.01 |
|  | Mother | 0.26 | 17 | 7.25 | 1.71 | 5.98 | 7.13 | 8.52 |
|  | Daughter | 0.32 | 21 | 7.46 | 3.32 | 5.03 | 6.98 | 8.01 |
|  | Other | 0.42 | 27 | 6.58 | 1.69 | 5.37 | 6.21 | 7.06 |
| *cln2Δ* | Total |  | 116 | 5.94 | 1.37 | 5.14 | 5.87 | 6.64 |
|  | Mother | 0.20 | 23 | 6.13 | 1.64 | 4.76 | 6.11 | 6.94 |
|  | Daughter | 0.16 | 19 | 6.17 | 1.91 | 5.20 | 5.70 | 6.74 |
|  | Other | 0.64 | 74 | 5.82 | 1.09 | 5.15 | 5.87 | 6.62 |
| *cln1Δcln2Δ* | Total |  | 70 | 6.00 | 1.86 | 4.89 | 5.71 | 6.87 |
|  | Mother | 0.17 | 12 | 6.13 | 1.68 | 5.11 | 6.00 | 6.88 |
|  | Daughter | 0.50 | 35 | 6.10 | 2.26 | 4.89 | 5.45 | 6.87 |
|  | Other | 0.33 | 23 | 5.79 | 1.19 | 4.83 | 5.63 | 6.87 |
| *sic1Δ* | Total |  | 100 | 5.91 | 1.22 | 5.15 | 5.65 | 6.56 |
|  | Mother | 0.21 | 21 | 6.09 | 1.41 | 5.10 | 6.13 | 6.89 |
|  | Daughter | 0.46 | 46 | 6.00 | 1.29 | 5.25 | 5.75 | 6.54 |
|  | Other | 0.33 | 33 | 5.66 | 0.97 | 5.05 | 5.50 | 6.11 |
| *sic1Δswi4Δ* | Total |  | 73 | 7.51 | 3.06 | 5.67 | 6.50 | 8.76 |
|  | Mother | 0.34 | 25 | 7.82 | 3.30 | 5.69 | 7.10 | 8.83 |
|  | Daughter | 0.38 | 28 | 8.12 | 3.33 | 5.80 | 6.83 | 9.21 |
|  | Other | 0.27 | 20 | 6.26 | 1.89 | 4.94 | 5.94 | 7.34 |
| *sic1Δmbp1Δ* | Total |  | 98 | 7.95 | 2.98 | 6.16 | 7.01 | 8.78 |
|  | Mother | 0.11 | 11 | 9.35 | 4.61 | 5.86 | 8.34 | 12.12 |
|  | Daughter | 0.44 | 43 | 7.82 | 2.62 | 6.24 | 7.09 | 8.22 |
|  | Other | 0.45 | 44 | 7.73 | 2.80 | 6.03 | 6.77 | 8.88 |
| *sic1Δwhi5Δ* | Total |  | 45 | 7.96 | 4.21 | 5.79 | 6.85 | 8.46 |
|  | Mother | 0.13 | 6 | 10.31 | 8.99 | 5.94 | 6.83 | 9.10 |
|  | Daughter | 0.20 | 9 | 8.35 | 3.35 | 5.89 | 7.06 | 10.51 |
|  | Other | 0.67 | 30 | 7.38 | 2.88 | 5.54 | 6.64 | 8.11 |
| *sic1Δcln2Δ* | Total |  | 105 | 8.15 | 3.72 | 6.00 | 7.00 | 8.90 |
|  | Mother | 0.18 | 19 | 7.46 | 3.40 | 5.82 | 6.83 | 7.64 |
|  | Daughter | 0.23 | 24 | 8.89 | 4.12 | 5.77 | 7.85 | 10.48 |
|  | Other | 0.59 | 62 | 8.08 | 3.66 | 6.02 | 7.19 | 8.70 |
| *sic1Δ cln1Δ*  *cln2Δ* | Total |  | 130 | 14.35 | 11.20 | 7.08 | 11.36 | 18.28 |
|  | Mother | 0.32 | 41 | 15.99 | 14.67 | 7.14 | 11.67 | 18.95 |
|  | Daughter | 0.39 | 51 | 13.18 | 7.99 | 7.28 | 11.28 | 17.04 |
|  | Other | 0.29 | 38 | 14.15 | 10.65 | 6.91 | 9.91 | 18.76 |
| *clb5Δ* | Total |  | 145 | 8.63 | 3.35 | 6.54 | 7.59 | 9.58 |
|  | Mother | 0.19 | 28 | 8.88 | 3.42 | 6.20 | 7.97 | 10.97 |
|  | Daughter | 0.26 | 37 | 8.91 | 3.40 | 6.87 | 7.81 | 9.91 |
|  | Other | 0.55 | 80 | 8.42 | 3.33 | 6.50 | 7.29 | 9.41 |
| *clb6Δ* | Total |  | 135 | 6.79 | 2.16 | 5.23 | 6.19 | 7.75 |
|  | Mother | 0.13 | 18 | 6.62 | 1.98 | 5.16 | 6.37 | 7.62 |
|  | Daughter | 0.33 | 45 | 7.57 | 2.71 | 5.52 | 7.08 | 9.06 |
|  | Other | 0.53 | 72 | 6.35 | 1.65 | 5.21 | 6.09 | 6.76 |
| *clb5Δclb6Δ* | Total |  | 89 | 11.05 | 5.07 | 7.64 | 9.57 | 12.87 |
|  | Mother | 0.38 | 34 | 11.74 | 5.98 | 7.64 | 8.88 | 15.41 |
|  | Daughter | 0.37 | 33 | 10.68 | 4.04 | 7.61 | 9.12 | 13.22 |
|  | Other | 0.25 | 22 | 10.54 | 5.02 | 7.51 | 9.67 | 11.42 |
