# Supplementary material for: Design Principles of the Yeast G1/S Switch
Source: PLoS Biol. 2013 Oct 1;11(10):e1001673. doi: 10.1371/journal.pbio.1001673 (PMC3794861; doi:10.1371/journal.pbio.1001673)
Supplement: Table S4 — Statistical tests of Sic1* half-life distribution. (DOC) [file pbio.1001673.s009.doc]

**Table S4.**  **Statistical tests of Sic1* half-life distribution. (Supplement for Figure 3)**

|  | **p values (t test vs WT)** | **p values (Mann Whitney test vs WT)** | **ANOVA+Dunnett's test** | **Kruskal-Wallis+Dunn test** |
| --- | --- | --- | --- | --- |
| *swi4Δ* | 0.7469 | 0.1613 | 1 | 0.3075 |
| *cln1Δcln2Δ* | 0.0636 | 0.0033 | 0.922 | 0.0203 |
| *clb6Δ* | 0.0335 | 0.8043 | 0.668 | 0.5795 |
| *whi5Δ* | 0.0041 | 0.1385 | 0.402 | 0.174 |
| *cln2Δ* | 0.004 | 0.0008 | 0.711 | 0.011 |
| *mbp1Δ* | 0.0016 | 0.0417 | 0.28 | 0.0745 |
| *clb5Δ** | < 0.0001 | < 0.0001 | < 0.001 | < 0.00001 |
| *clb5Δclb6Δ** | < 0.0001 | < 0.0001 | < 0.001 | < 0.00001 |
