# Supplementary material for: Design Principles of the Yeast G1/S Switch
Source: PLoS Biol. 2013 Oct 1;11(10):e1001673. doi: 10.1371/journal.pbio.1001673 (PMC3794861; doi:10.1371/journal.pbio.1001673)
Supplement: Table S5 — Statistical tests of Sic1* half-life distribution under genetic perturbations (with and without DNFBL). (DOC) [file pbio.1001673.s010.doc]

**Table S5. Statistical tests of Sic1* half-life distribution under genetic perturbations (with and without DNFBL). (Supplement for Figure 3)**

| vs *WT* | *swi4Δ* | *mbp1Δ* | *whi5Δ* | *cln2Δ* | *cln1Δcln2Δ* |
| --- | --- | --- | --- | --- | --- |
| ANOVA+Dunnett's test | 0.9994 | 0.0164 | 0.0396 | 0.1849 | 0.4833 |
| Kruskal-Wallis+Dunn test | 0.1693 | 0.087 | 0.2377 | 0.0025 | 0.005 |
|  |  |  |  |  |  |
| vs *sic1Δ* | *sic1Δswi4Δ* | *sic1Δmbp1Δ* | *sic1Δwhi5Δ* | *sic1Δcln2Δ* | *sic1Δcln1Δcln2Δ* |
| ANOVA+Dunnett's test | < 1e-9 | < 1e-9 | 2.66E-09 | < 1e-9 | < 1e-9 |
| Kruskal-Wallis+Dunn test | 0.0002 | < 0.00001 | 0.0003 | < 0.00001 | < 0.00001 |
