# Supplementary material for: Design Principles of the Yeast G1/S Switch
Source: PLoS Biol. 2013 Oct 1;11(10):e1001673. doi: 10.1371/journal.pbio.1001673 (PMC3794861; doi:10.1371/journal.pbio.1001673)
Supplement: Table S6 — Sic1* half-life under environmental perturbations (with and without DNFBL). (DOC) [file pbio.1001673.s011.doc]

**Table S6. Sic1* half-life under environmental perturbations (with and without DNFBL). (Supplement for Figure 3)**

|  | Number of cells | Mean | s.d. | 1st quartile | Median | 3rd quartile |
| --- | --- | --- | --- | --- | --- | --- |
| *WT* | 143 | 6.37 | 0.99 | 5.71 | 6.44 | 6.92 |
| *WT* 37°C | 101 | 5.94 | 1.53 | 4.94 | 5.76 | 6.40 |
| *WT* Tunicamycin | 79 | 6.82 | 2.41 | 5.24 | 6.21 | 7.81 |
| *WT* 0.5M KCl | 75 | 7.15 | 1.90 | 6.04 | 6.70 | 7.41 |
| *WT* 1M KCl | 31 | 9.43 | 4.02 | 6.86 | 8.26 | 10.38 |
|  |  |  |  |  |  |  |
| *sic1Δ* | 100 | 5.91 | 1.22 | 5.15 | 5.65 | 6.56 |
| *sic1Δ* 37°C | 95 | 6.18 | 1.95 | 4.86 | 5.68 | 6.96 |
| *sic1Δ* Tunicamycin | 98 | 9.27 | 6.74 | 5.67 | 7.40 | 10.32 |
| *sic1Δ* 0.5M KCl | 61 | 10.47 | 4.69 | 7.37 | 8.28 | 12.95 |
| *sic1Δ* 1M KCl | 66 | 21.12 | 18.16 | 12.13 | 16.27 | 23.44 |
