# Supplementary material for: Design Principles of the Yeast G1/S Switch
Source: PLoS Biol. 2013 Oct 1;11(10):e1001673. doi: 10.1371/journal.pbio.1001673 (PMC3794861; doi:10.1371/journal.pbio.1001673)
Supplement: Table S7 — Statistical tests of Sic1* half-life distribution under environmental perturbations (with and without DNFBL). (DOC) [file pbio.1001673.s012.doc]

**Table S7. Statistical tests of Sic1* half-life distribution under environmental perturbations (with and without DNFBL). (Supplement for Figure 3)**

| vs *WT* | *WT* 37°C | *WT*  Tunicamycin | *WT* 0.5M KCl | *WT* 1M KCl |
| --- | --- | --- | --- | --- |
| ANOVA+Dunnett | 0.255 | 0.26 | 0.787 | < 1e-4 |
| t test | 0.009 | 0.0482 | 0.0736 | < 0.0001 |
| Kruskal-Wallis+Dunn | 0.0006 | 0.9183 | 0.1804 | < 0.00001 |
| Mann-Whitney | 0.0001 | 0.9547 | 0.1272 | < 0.0001 |
|  |  |  |  |  |
| vs *sic1Δ* | *sic1Δ* 37°C | *sic1Δ* Tunicamycin | *sic1Δ* 0.5M KCl | *sic1Δ* 1M KCl |
| ANOVA+Dunnett | 0.9981 | 0.01453 | 0.00234 | < 0.001 |
| t test | 0.2331 | < 0.0001 | < 0.0001 | < 0.0001 |
| Kruskal-Wallis+Dunn | 0.7279 | < 0.00001 | < 0.00001 | < 0.00001 |
| Mann-Whitney | 0.997 | < 0.0001 | < 0.0001 | < 0.0001 |
