# Supplementary material for: Design Principles of the Yeast G1/S Switch
Source: PLoS Biol. 2013 Oct 1;11(10):e1001673. doi: 10.1371/journal.pbio.1001673 (PMC3794861; doi:10.1371/journal.pbio.1001673)
Supplement: Table S8 — Dynamics of promoters CLN2pr/CLB5pr in different strains. (DOC) [file pbio.1001673.s013.doc]

**Table S8. Dynamics of promoters CLN2pr/CLB5pr in different strains. (Supplement for Figure 3)**

|  | promoter start (min) | s.d. | promoter start to promoter peak | s.d. | Number of cells |
| --- | --- | --- | --- | --- | --- |
|  | (from MCM entering nucleus) |  | (min) |  |  |
| *WT CLN2pr* | 5.52 | 3.91 | 16.61 | 2.2 | 100 |
| *mbp1Δ CLN2pr* | 8.44 | 7.39 | 21.63 | 4.27 | 100 |
| *swi4Δ CLN2pr* | 8.63 | 5.78 | 31.23 | 11.05 | 100 |
| *WT CLB5pr* | 7.03 | 8.78 | 19.8 | 5.03 | 121 |
| *mbp1Δ CLB5pr* | 6.91 | 8.8 | 22.6 | 8.88 | 104 |
| *swi4ΔCLB5pr* | 8.55 | 8.35 | 20.83 | 8.17 | 102 |
