# Supplementary material for: Design Principles of the Yeast G1/S Switch
Source: PLoS Biol. 2013 Oct 1;11(10):e1001673. doi: 10.1371/journal.pbio.1001673 (PMC3794861; doi:10.1371/journal.pbio.1001673)
Supplement: Text S1 — Extended experimental procedures. (PDF) [file pbio.1001673.s014.pdf]

Supporting Text S1

Extended Experimental Procedures

Chromosome Loss

We accessed the impact of different Sic1 mutants on genome stability by examining the mitotic loss rate of a minichromosome, pDK243, with and without extra ARSs (Autonomous Replicating Sequence) in wild-type and all various SIC1 mutants. pDK243 is an artificial circular chromosome containing a single origin of replication(*ARS1*), a centromere(*CEN3*), and a *ADE* marker[1,2]. Note that the rate of improper segregation for the minichromosome is 200-fold higher than observed for a normal chromosome[2].

Since minichromosome loss can stem either from replication failure or from unequal partitioning to daughter cells, and only if it is due to replication defects should extra origins rescue its loss, we also measured the loss rate of pDK368-7, which is a derivative of pDK243 that contains eight tandem copies of ARSs. The addition of multiple ARS inserts suppressed its loss in Sic1 mutants, suggesting these Sic1 mutants are defective in initiation of DNA replication (Fig.S4F, lighter bars).

Mitotic loss rate were determined in WT and Sic1 mutant cells with *ade2Δade3Δ* background, carrying pDK(*ADE3*) plasmids with one or eight ARSs [7] using the formula: loss rate=1-10^m, where m=[ln(Ft)-ln(Fi)]/n, F(i) being the initial and F(t) the fraction of cells containing the plasmid at time point t after n doublings. The fraction of plasmid-containing cells was determined by counting the red colonies (*ADE3*) (typically ~1000 cells were counted). Cells were grown exponentially throughout the experiments, and doubling time was measured at the same time to calculate n. For each single mutant, four colonies were picked; for each colony, four time points were taken to fit the loss rate. An example of the loss rate fitting is shown below:

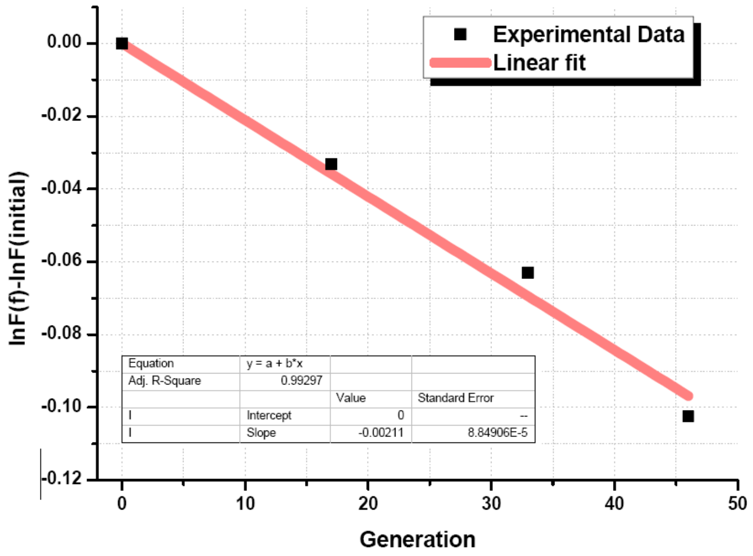

Note that although we observed different phosphorylation site contributes to Sic1 degradation differently and resulting various level of genome instability, it seems still premature to speculate on the molecular explanation for this difference between mutants.

FACS.

For flow cytometry analyses, cells were fixed in 70% ethanol, washed with 50 mM Tris.HCl (pH7.5), treated with 1 mg/ml RNase A at 37°C for 2hrs, then added 20mg/ml Proteinase K for 1 hr at 55°C, washed with 50mM Tris.HCl, and then resuspended by FACS buffer [200mM Tris.HCl pH7.5, 200mM NaCl, 78mM $MgCl_2$], stained with 4 µg/ml propidium iodide, and analyzed in a FACScan flow cytometer. About Hundred thousand cells were analyzed for each sample.

Image analysis

Images from time-lapsed fluorescence microscopy were analyzed using ImageJ (http://rsbweb.nih.gov/ij/) and custom software written in Matlab. The analysis process is as follows:

Cell selection

Cells are selected from the bright field image based on whether the cell is growing (bud formation) and whether the cell is in focus etc. Fluorescence signals are used as a criterion only to reject cells that have no visible signal. There is no bias towards strong or weak fluorescence signals as long as they are visible. An example of the cell selection is shown below (selected cells are labeled with a green cross)

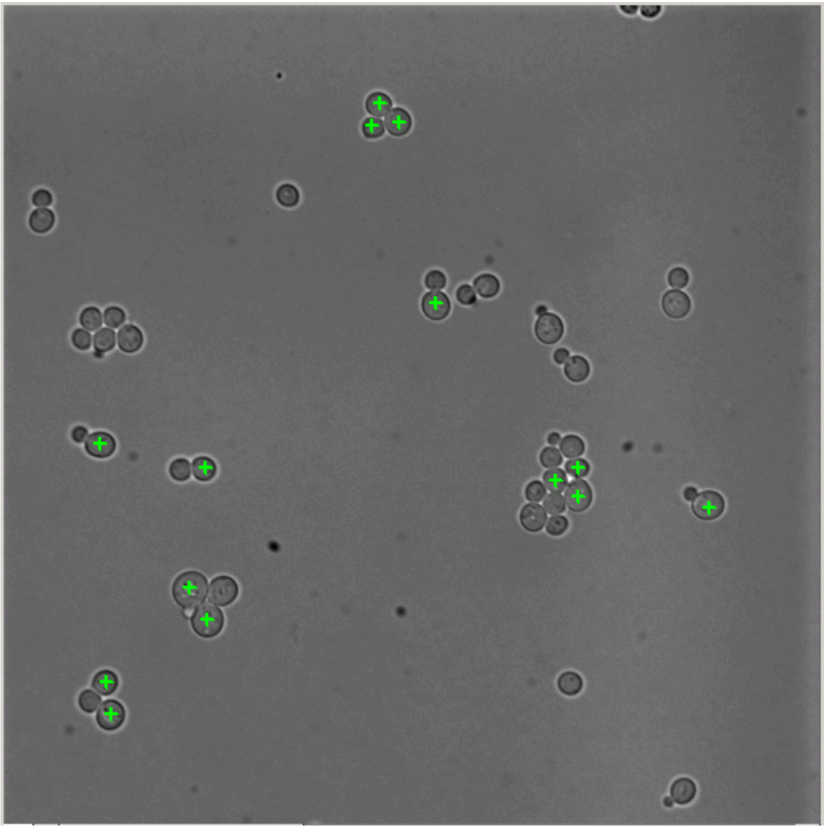

Cell Segmentation

After cells are selected, segmentation is done automatically from the time series of bright field images using the parameters provided by the user. For example, user can specify the maximum and minimum cell size. Segmenting cells from the bright field images ensures that the segmented cells are not affected by their fluorescence signal. The bright field images are taken slightly out of focus to create a bright edge surrounding individual cells. These bright edges are traced by the software and returned as the boundary between cells and non-cells. Each cell is numbered for

further analysis. An example of cell segmentation is shown below (Cell enclosed red lines and that trace the cell boundaries. Individual cells are assigned with a unique number, shown in cyan)

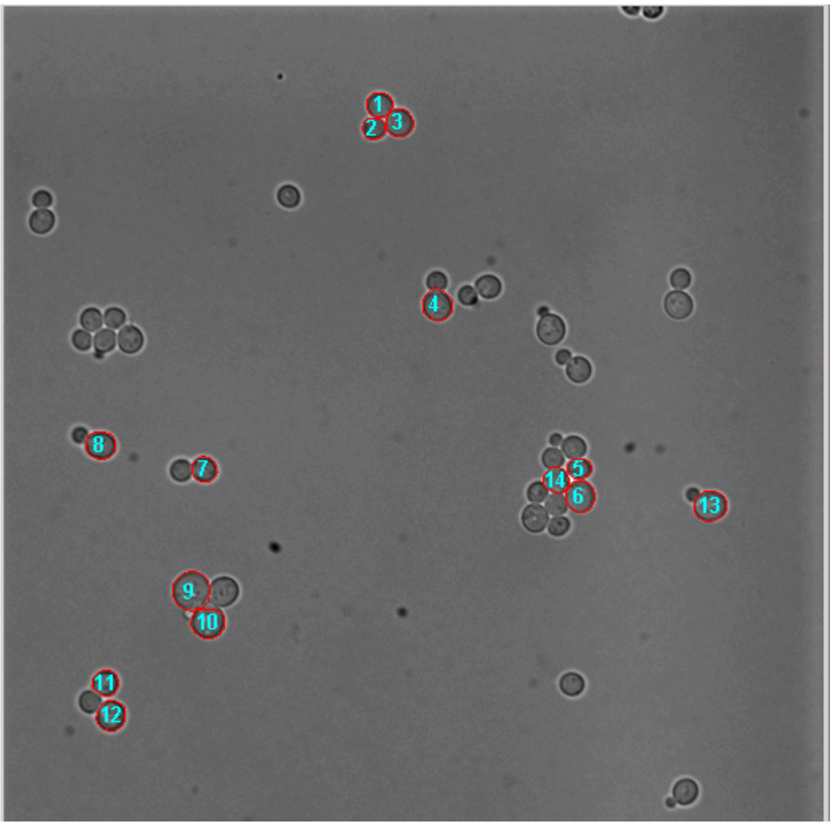

The software also allow manual correction for the segmented cells, as shown below. (Left panel: a blue polygon is used to interact with user; Right panel: After modification.)

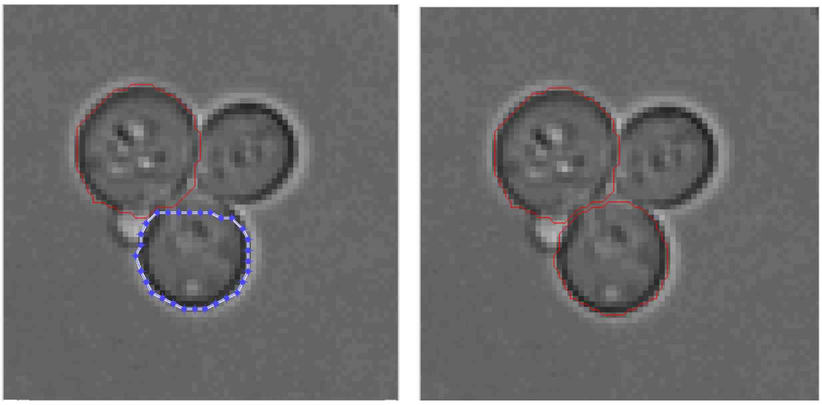

Nucleus Segmentation

Nucleus Segmentation is done, when necessary, after cells are segmented. Nuclei are segmented from the fluorescently labeled nuclear images. The software locates the edge between the bright nucleus and dark background within each cell and returns the bright area as the nucleus. An example of nucleus segmentation is shown below (segmented nuclei are enclosed by yellow lines)

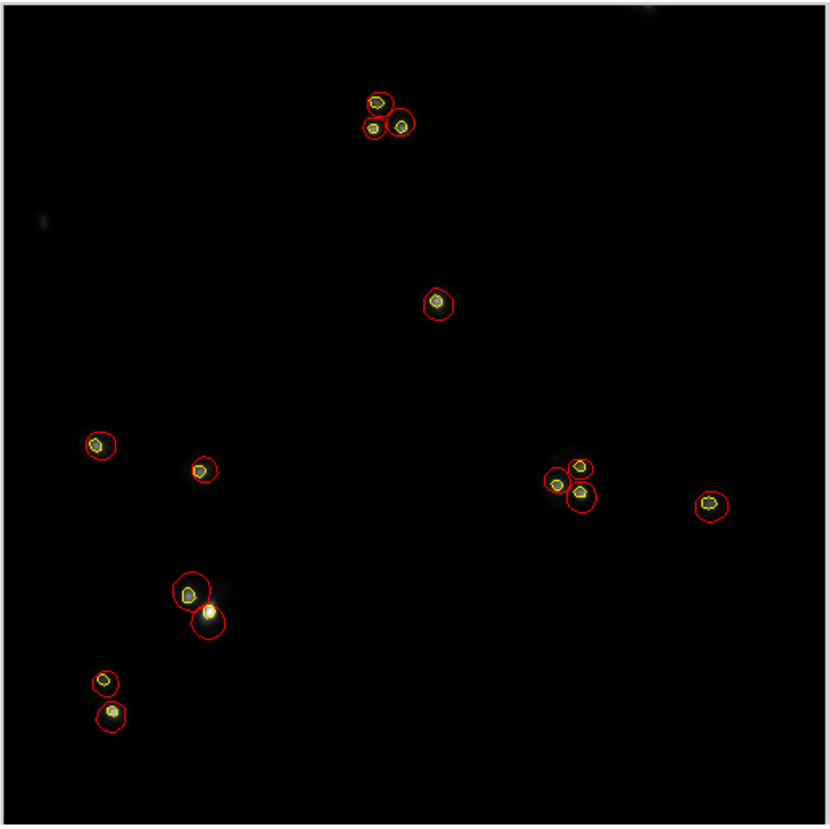

Background subtraction

Background subtraction for fluorescent images was performed using the background subtraction function from ImageJ.

Export data

Signal intensity from the background subtracted fluorescent images for the segmented cells were then exported to tab delimited text files using the custom software. For Sic1 reporter, we found the 3x3 square of pixels within each cell that has the maximum signal intensity and exported its mean. For endogenous Sic1, we exported the mean nuclear signal intensity for Sic1 and the nuclear label for each cell.

Data analysis

Half-life analysis

Fluorescence signals from endogenous Sic1 or Sic1 reporter were extracted from the images as described in the above section. We fitted an exponential function to the signal intensity data to get the decay rate and in turn get the half lives of the proteins (Figure 2C, 2F, and Figure 3D-3F).

For Sic1reporter, we fitted the function directly onto the mean reporter fluorescence signal from the maximum 3x3 square. For endogenous Sic1, we first divided the mean nuclear Sic1 intensity by the mean nuclear label intensity and fitted the function onto the resulting values.

The function we fitted to the fluorescence signal is

$$y = Ae^{-\lambda t} + C \qquad (1)$$

where y is the time series of fluorescence signal. However, since this function will cause a bias towards larger values, we also fit the signal to this form of the function.

$$\log(y - C) = \log(A) - \lambda t \qquad (2)$$

To fit using Eq 2, we first found the constant C that gives the straightest line in the log domain when subtracted from the data. Then we fitted a straight line to the log subtracted data and the absolute value of the slope is the decay rate.

An example of fitting the exponential function to the measured fluorescent signal is shown below (Left panel: original data in blue x's, the fitting range is between the yellow and green dash lines, the fitted line is in red. Right panel: log C-subtracted data in blue x's (and magenta circles for values smaller than C).

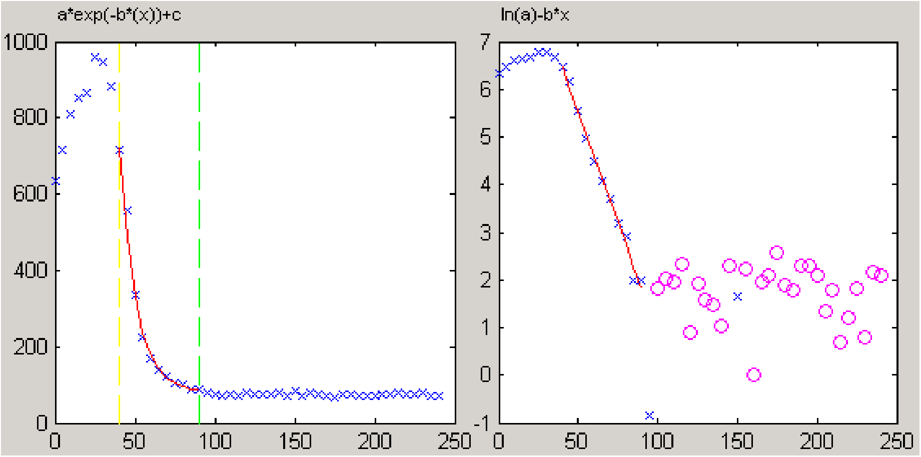

The potential correlation between the Sic1 half life and the number of points used to fit was checked, and no correlation was found, shown below.

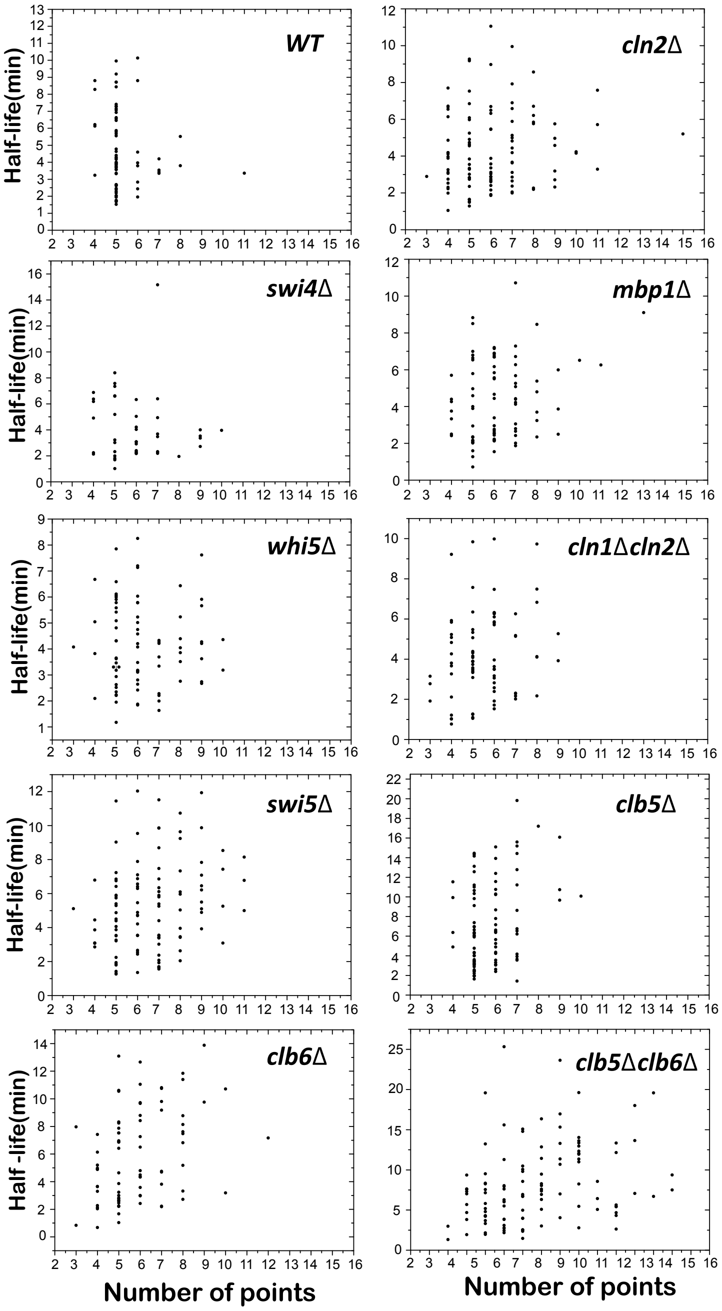

Promoter activity analysis

We measured the dynamics of *CLN2* and *CLB5* promoters by monitoring a destabilized GFP controlled by the promoters of *CLN2* and *CLB5*. As a timing reference we also monitored an MCM marker in the same cells. The MCM marker enters the nucleus at the end of M phase and exits the nucleus during G1 to S transition, therefore, the change in nuclear localization of MCM marker signal tells us at which cell cycle phase the cells are in. The localization of the signal is quantified as the standard deviation of the fluorescent signal across the cell (the more it is localized, the large the standard deviation is). Since we were measuring the intensity of the GFP over time and the fluorescent signal is delayed due to the time for maturation. We de-convoluted the maturation time from the GFP signal to get the dynamics of total GFP (matured and unmatured). We then de-convoluted the degradation of GFP to get the dynamics of the promoters.

Briefly, assume the following model for GFP protein production and maturation,

$$\frac{d}{dt}GFPT = P(t) - \gamma GFPT \qquad (3)$$

$$\frac{d}{dt}GFP^{*} = k(GFPT - GFP^{*}) - \gamma GFP^{*} \qquad (4)$$

where GFPT is the total GFP, GFP* is the matured GFP, *P(t)* is the promoter activity, γ (=log(2)/40) is the degradation rate of GFP[3], k (= 0.067) is the maturation rate of GFP. After some conversions, the equation for promoter activity is

$$P(t) = \frac{1}{k}\frac{d^2}{dt^2}GFP^{*} + \left(1 + 2\frac{\gamma}{k}\right)\frac{d}{dt}GFP^{*} + \left(\gamma + \frac{\gamma^2}{k}\right)GFP^{*} \qquad (5)$$

This is a function of matured GFP and can be calculated from the measured fluorescent signal. To get the first and second derivatives of GFP*, we fitted a smoothing spline onto the GFP fluorescent signal and differentiate the spline function. We used the data from the MCM marker directly without any de-convolution since the marker is expressed constitutively and we were only concerned about its localization (in and out of nucleus).

An example of promoter activity is shown below (In the first plot, green curve is the smoothing spline fitted onto the measured fluorescent signal (x's) and the red curve is the smoothing spline fitted onto the standard deviation of the MCM marker signal (o's). In the second plot, the green curve is the promoter activity calculated from Eq 5 and the blue curve is the second derivative of the green curve. In the third plot, the green curve is the total GFP after de-convoluting the maturation time and the blue curve is the second derivative of the green curve. In the last plot, the green curve is the second derivative of the green smoothing spline in the first plot and the red curve is the second derivative of the red smoothing spline in the first plot. The first timing measurement, from MCM enters nucleus to promoter activation, is the time from the red dash line to the blue dash line. The second timing measurement, from promoter activation to promoter reaches peak level, is the time from the blue dash line to the green dash line.)

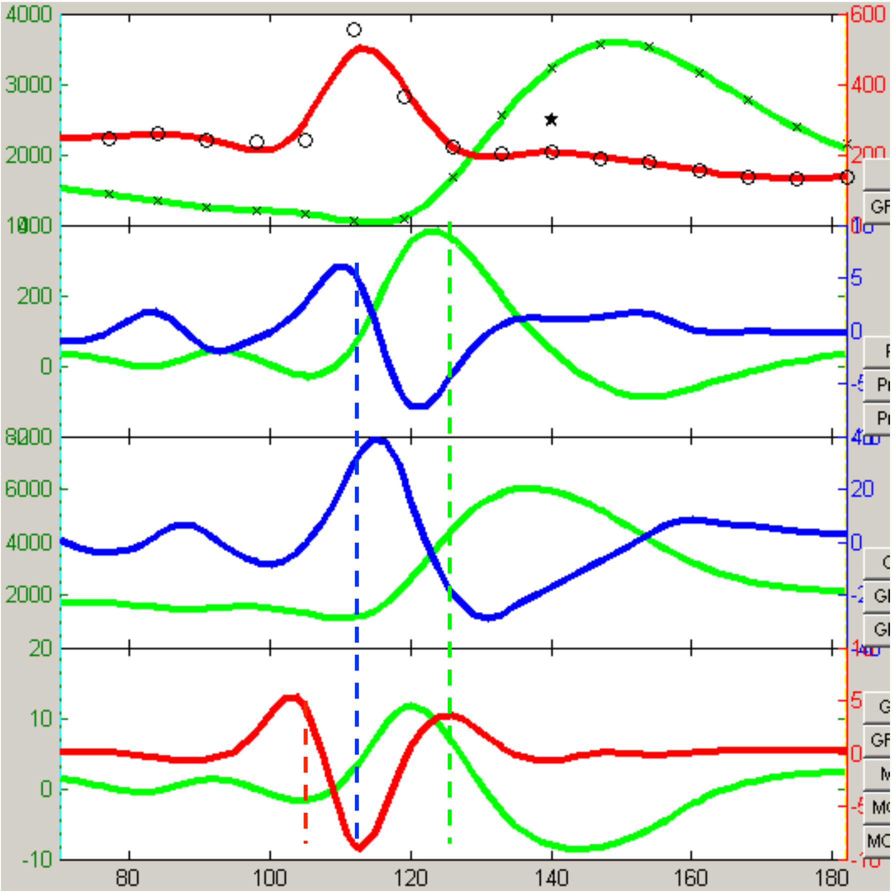

We measured the following timing information for the promoter activity,

1) The time between MCM marker entering the nucleus to the activation of the promoter.

We calculated the difference between the time when the second derivative of MCM marker reaches a local maximum to the time when the second derivative of the promoter reaches a local maximum. (From red to blue dash line).

2) The time between promoter activation to peak promoter activity.

We calculated the difference between the time when the second derivative of the promoter reaches a local maximum and the time when the promoter activity reaches the peak. (From blue to green dash line).

Time-lapse microscopy pre-experiments

Cells growing exponentially in synthetic liquid medium were seeded onto thin 1.5%-2% agrose slabs in the same medium. A typical time-lapse experiment was limited by cell stacking effects to between six and nine hours. Multiple different positions were followed simultaneously.

Cells growing under time-lapse conditions, but without illumination for fluorescence detection, showed exponential growth throughout the course of image acquisition, and showed a comparable doubling time (~80mins) as in liquid media at 30°C (SD medium

Doubling time in SD medium without illumination for fluorescence detection —two 6hrs experiments

		Mean(min)	s.d.	Number of cells
Mother	Bright Field(1)	71.34	9.60	71
	Bright Field(2)	72.67	12.70	60
Daughter	Bright Field(1)	96.91	20.87	47
	Bright Field(2)	99.88	18.85	43
All	Bright Field(1)	81.53	19.61	118
	Bright Field(2)	84.03	20.53	103

The Doubling time was measured as below: The mother doubling time is measured by the mother bud-to-bud interval, labeled as MB1-MB2, and the daughter doubling time is measured by the daughter bud-to-bud interval, labeled as MB1-DB1[9]

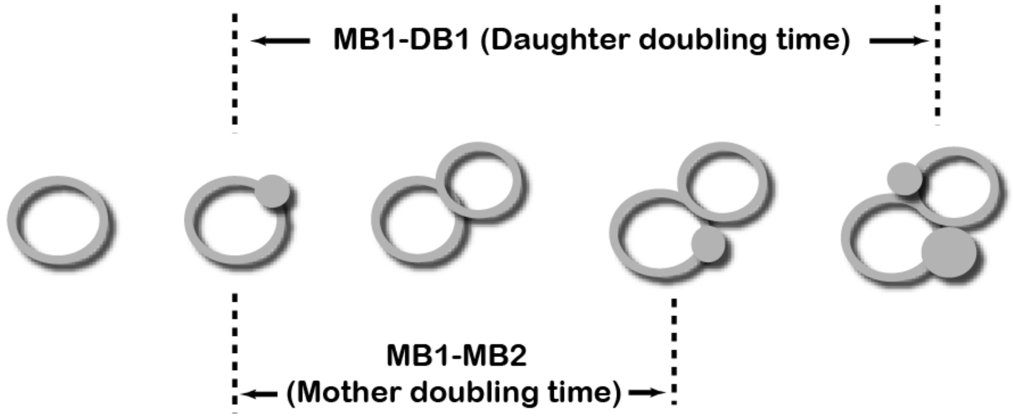

Endogenous Sic1 pre-experiments

The growth of cells in selective medium (SC ura- his-) under time-lapse conditions, but without illumination for fluorescence detection was checked, and showed a comparable doubling time as in liquid media at 30°C

Doubling time in SC ura- his- medium without illumination for fluorescence detection—two 6hrs experiment.

		Mean(min)	s.d.	Number of cells
All	BF-5min interval	108.05	27.17	82
	BF-1 min interval	107.63	28.34	78

Detection of mCherry fluorescence was done with 5ms illumination and detection of GFP fluorescence was done with 7ms illumination. When illuminated every 3 min, no appreciable effects on the growth rate were observed (the doubling time varies within 10%). When the illumination interval is less than 3 min, longer doubling times were observed (SC ura- his-medium), as shown below

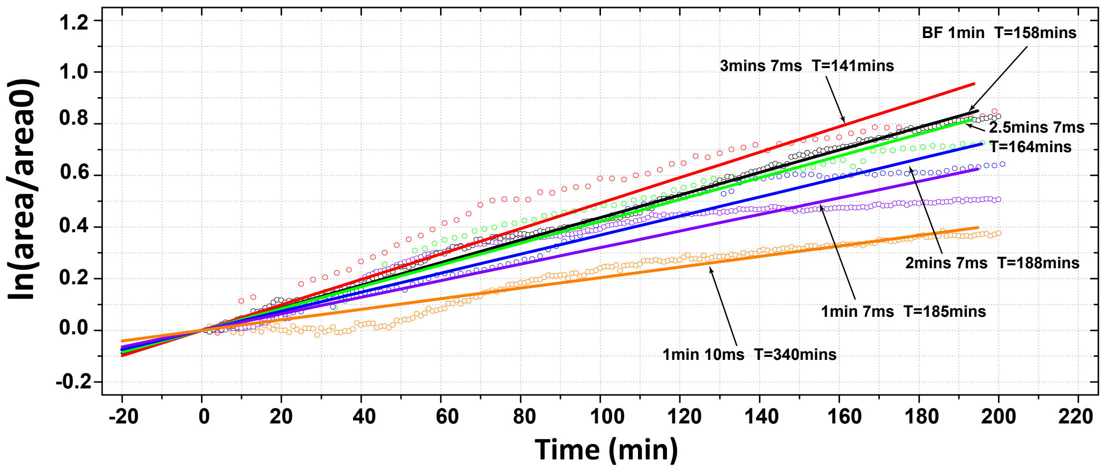

The interval between the exposure, the duration of the exposure, and the doubling time of the cell cluster are indicated for each growth curve. Cell areas were used to fit the growth rate."#$"example of cell area measurement is shown below (Left panel: phase contrast image of a cell cluster grown under time-lapse exposure. Right panel: cell area obtained by ImageJ.)

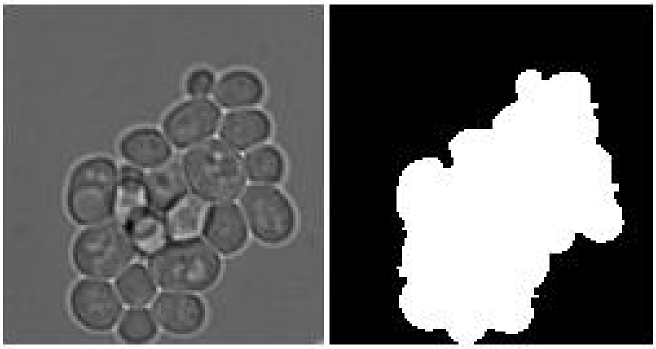

Doubling time of cells at varying intervals between and duration of the exposure.

		Mean(min)	s.d.	Number of cells
Mother	BF-1min	100.67	28.87	48
	3mins-7ms	112.55	19.52	20
	2.5mins-7ms	114.89	21.67	23
	2mins-7ms	137.33	32.27	15
	1min-7ms	142.67	67.87	3
	1min-10ms	241.00	0	1
Daughter	BF-1min	118.77	23.93	30
	3mins-7ms	120.4	31.13	15
	2.5mins-7ms	119.58	20.91	12
	2mins-7ms	152.29	12.41	7
	1min-7ms	-	0	0
	1min-10ms	-	0	0

Total	BF-1min	107.63	28.34	78
	3mins-7ms	114.34	33.12	35
	2.5mins-7ms	116.50	21.22	35
	2mins-7ms	142.09	38.09	22
	1min-7ms	142.67	67.87	3
	1min-10ms	241	0	1

As for the endogenous Sic1, short half-life requires a high temporal resolution. The degradation dynamics of Sic1 at varying time intervals are the same.although some intervals have a slightly longer doubling time, as shown below.

Sic1 degradation in single cells at varying time intervals. (*WT*)

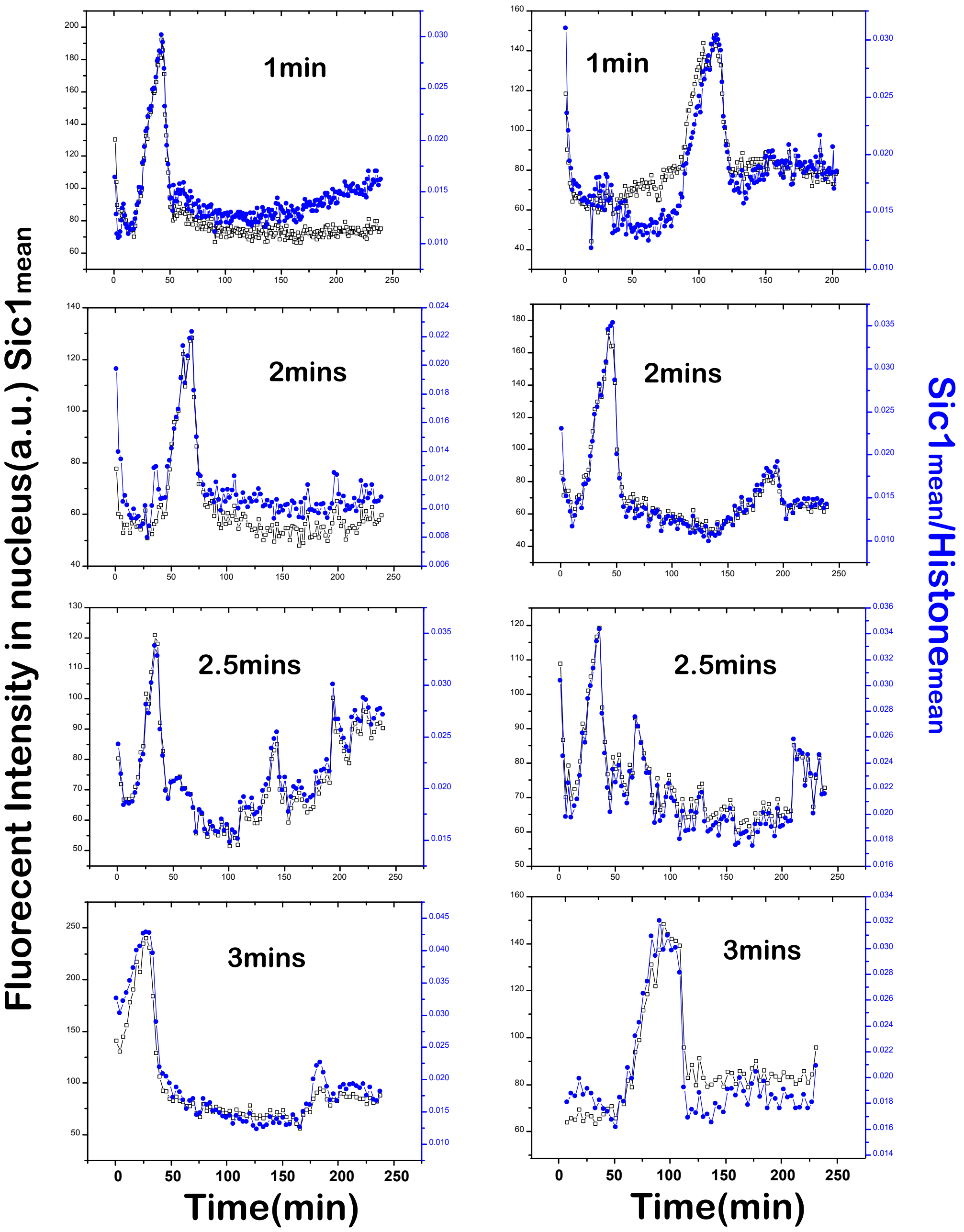

Sic1 half-life at varying time intervals.

	Mean	s.d.	1st Quartile	Median	3rd Quartile	Number of cells
1min	4.60808	2.30109	2.68556	3.93315	6.21718	73
2mins	3.68975	2.13113	2.42205	3.15774	3.93443	21
2.5mins	3.36382	1.05579	2.65924	3.18787	3.88206	25
3mins	4.56732	1.93375	3.29281	3.70622	6.21626	35

Sic1 half-life at varying time intervals in *clb5Δ clb6Δ* cells. Each dot represents the half-life from a single cell.

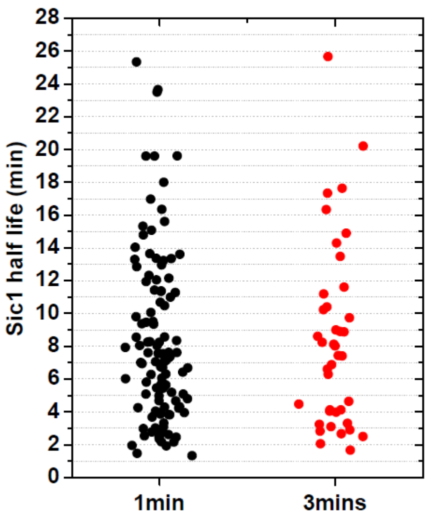

Therefore, we used 1min interval throughout for endogenous Sic1 experiments to gain more dynamic information.

Sic1* pre-experiments

Detection of mCherry fluorescence was by 1s illumination and no appreciable effect on the doubling time was observed when illuminating every 5mins (SC ura- medium), indicating that the condition used in Sic1* experiments do not induce significant toxicity or perturbations to cell cycle. Data is shown below (Left: The doubling time distribution of mother cells with and without 1s mCherry exposure. Right: Scatter

plots of paired bud-to-bud intervals for mothers and daughters with and without fluorescent exposure.)

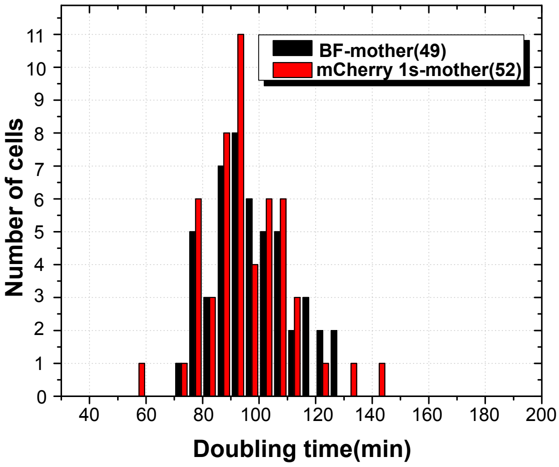

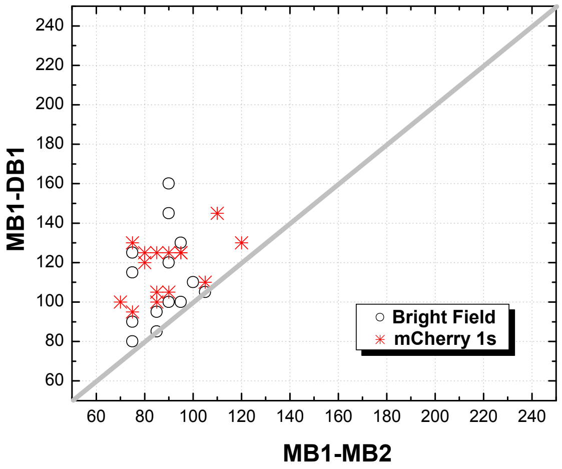

Doubling time with and without mCherry exposure in SC ura- medium.

		Mean(min)	s.d.	Num.
Mother	Bright Field	94.90	14.09	49
	mCherry 1s	92.60	14.93	52
Daughter	Bright Field	117.67	23.14	15
	mCherry 1s	117.67	14.25	15
All	Bright Field	100.23	19.10	64
	mCherry 1s	98.21	18.06	67

Promoter pre-experiments

Detection of mCherry fluorescence was done with 1s illumination and detection of GFP fluorescence was done with 1s illumination, and no appreciable effect on the doubling time was observed when illuminating every 7mins (SC ura- his- medium)

Doubling time of different strains.

		Mean (min)	s.d.	Number of

				cells
Mother	CLN2pr	109.15	33.93	54
	CLB5pr	100.25	19.58	56
	BF	99.21	26.14	52
Daughter	CLN2pr	131.5	32.35	28
	CLB5pr	127.48	23.46	33
	BF	123.24	26.25	33
All	CLN2pr	116.78	34.87	82
	CLB5pr	110.35	24.79	89
	BF	108.54	28.57	85

1. E. Hogan, D. Koshland, Addition of extra origins of replication to a minichromosome suppresses its mitotic loss in cdc6 and cdc14 mutants of Saccharomyces cerevisiae, Proc. Natl. Acad. Sci. U.S.A 89, 3098-3102 (1992).
2. D. Koshland, J. C. Kent, L. H. Hartwell, Genetic analysis of the mitotic transmission of minichromosomes, Cell 40, 393-403 (1985).
3. Bean JM, Siggia ED, Cross FR (2006) Coherence and timing of cell cycle start examined at single-cell resolution. Mol Cell 21: 3–14. doi:10.1016/j.molcel.2005.10.035.
