# Supplementary material for: Design Principles of the Yeast G1/S Switch
Source: PLoS Biol. 2013 Oct 1;11(10):e1001673. doi: 10.1371/journal.pbio.1001673 (PMC3794861; doi:10.1371/journal.pbio.1001673)
Supplement: Text S2 — Stochastic simulation. (PDF) [file pbio.1001673.s015.pdf]

Supporting Text S2

Stochastic simulation

Equations for the results shown in Figure 2G and Supplementary Figure S1G, stochastic simulation is done under the Chemical Langevin Equations (CLE) framework (Gillespie, 2000; Gillespie 2007).

$$\frac{d}{dt}[\mathrm{Cln3}] = k_{n3}\, exp(0.0063t) - d_{n3}[\mathrm{Cln3}]$$

$$\frac{d}{dt}[\mathrm{Cln1/2}] = k_{n2,1} + \frac{k_{n2,sbf}[\mathrm{SBFA}] + k_{n2,mbf}[\mathrm{MBFP}]}{1 + K_{n2,sbf}[\mathrm{SBFA}] + K_{n2,mbf}[\mathrm{MBFP}]} - d_{n2}[\mathrm{Cln1/2}]$$

$$\frac{d}{dt}[\mathrm{Clb5/6}] = k_{b5,1} + \frac{k_{b5,sbf}[\mathrm{SBFA}] + k_{b5,mbf}[\mathrm{MBFP}]}{1 + K_{b5,sbf}[\mathrm{SBFA}] + K_{b5,mbf}[\mathrm{MBFP}]} - d_{b5}[\mathrm{Clb5/6}]$$

$$\frac{d}{dt}[\mathrm{Sic1}] = k_{c1}\left(\frac{K_{c1}}{K_{c1} + [\mathrm{Cln1/2}] + [\mathrm{Clb5/6_{active}}]}\right) - d_{c1,1}([\mathrm{Sic1}] - [\mathrm{Sic1P}]) - d_{c1,2}[\mathrm{Sic1P}]$$

$$\frac{d}{dt}[\mathrm{C5S}] = k_{as}([\mathrm{Sic1}] - [\mathrm{C5S}])[Clb5/6_{active}] - \left[k_{di} + d_{b5} + \left(1 - \frac{[\mathrm{Sic1P}]}{[\mathrm{Sic1}]}\right)d_{c1,1} + \frac{[\mathrm{Sic1P}]}{[\mathrm{Sic1}]}d_{c1,2}\right][\mathrm{C5S}]$$

$$\frac{d}{dt}[\mathrm{Sic1m}] = k_{c1m} - d_{c1m,1}([\mathrm{Sic1m}] - [\mathrm{Sic1mP}]) - d_{c1m,2}[\mathrm{Sic1mP}]$$

$$\frac{d}{dt}[\mathrm{Sic1P_1}] = V_{sic1}([\mathrm{Sic1}] - [\mathrm{Sic1PT}]) + k_{dp}[\mathrm{Sic1P_2}] - \left(V_{Sic1} + k_{dp} + d_{c1,1}\right)[\mathrm{Sic1P_1}]$$

$$\frac{d}{dt}[\mathrm{Sic1P_n}] = V_{sic1}[\mathrm{Sic1P_{n-1}}] + k_{dp}[\mathrm{Sic1P_{n+1}}] - \left(V_{Sic1} + k_{dp} + d_{c1,1}\right)[\mathrm{Sic1P_n}], n = 2 \cdots 5$$

$$\frac{d}{dt}[\mathrm{Sic1P_n}] = V_{Sic1}[\mathrm{Sic1P_{n-1}}] + k_{dp}[\mathrm{Sic1P_{n+1}}] - \left(V_{Sic1} + k_{dp} + d_{c1,2}\right)[\mathrm{Sic1P_n}], n = 6 \cdots 8$$

$$\frac{d}{dt}[\mathrm{Sic1P_9}] = V_{sic1}[\mathrm{Sic1P_8}] - \left(k_{dp} + d_{c1,2}\right)[\mathrm{Sic1P_9}]$$

$$\frac{d}{dt}[\mathrm{Sic1mP_1}] = V_{sic1m}([\mathrm{Sic1m}] - [\mathrm{Sic1mPT}]) + k_{dp}[\mathrm{Sic1mP_2}] - \left(V_{sic1m} + k_{dp} + d_{c1m,1}\right)[\mathrm{Sic1mP_1}]$$

$$\frac{d}{dt}[\mathrm{Sic1mP_n}] = V_{sic1m}[\mathrm{Sic1mP_{n-1}}] + k_{dp}[\mathrm{Sic1mP_{n+1}}] - \left(V_{sic1m} + k_{dp} + d_{c1m,1}\right)[\mathrm{Sic1mP_n}], n = 2 \cdots 5$$

$$\frac{d}{dt}[\mathrm{Sic1mP_n}] = V_{sic1m}[\mathrm{Sic1mP_{n-1}}] + k_{dp}[\mathrm{Sic1mP_{n+1}}] - \left(V_{sic1m} + k_{dp} + d_{c1m,2}\right)[\mathrm{Sic1mP_n}], n = 6 \cdots 8$$

$$\frac{d}{dt}[\mathrm{Sic1mP_9}] = V_{sic1m}[\mathrm{Sic1mP_8}] - \left(k_{dp} + d_{c1m,2}\right)[\mathrm{Sic1mP_9}]$$

$$[\mathrm{Sic1P}] = \sum_{n=6}^{9}[\mathrm{Sic1P_n}]$$

$$[\mathrm{Sic1PT}] = \sum_{n=1}^{9}[\mathrm{Sic1P_n}]$$

$$[\text{Sic1mP}] = \sum_{n=6}^{9} [\text{Sic1mP}_\text{n}]$$

$$[\text{Sic1mPT}] = \sum_{n=1}^{9} [\text{Sic1mP}_\text{n}]$$

$$[\text{Clb5/6}_\text{active}] = [\text{Clb5/6}] - [\text{C5S}]$$

$$V_{whi5} = \epsilon_{whi5,n3}[\text{Cln3}] + \epsilon_{whi5,n2}[\text{Cln1/2}] + \epsilon_{whi5,b5}[\text{Clb5/6}_\text{active}]$$

$$V_{mbf} = \epsilon_{mbf,n3}[\text{Cln3}] + \epsilon_{mbf,n2}[\text{Cln1/2}] + \epsilon_{mbf,b5}[\text{Clb5/6}_\text{active}]$$

$$V_{sic1} = \epsilon_{sic1,n2}[\text{Cln1/2}] + \epsilon_{sic1,b5}[\text{Clb5/6}_\text{active}]$$

$$V_{sic1m} = \epsilon_{sic1m,n2}[\text{Cln1/2}] + \epsilon_{sic1m,b5}[\text{Clb5/6}_\text{active}]$$

$$[\text{Whi5P}] = [\text{Whi5T}]\frac{V_{whi5}{}^{n_{whi5}}}{1 + V_{whi5}{}^{n_{whi5}}}$$

$$[\text{MBFP}] = [\text{MBFT}]\frac{V_{mbf}{}^{n_{mbf}}}{1 + V_{mbf}{}^{n_{mbf}}}$$

$$[\text{SBFA}] = [\text{SBFT}]\frac{K_{sbf}}{[Whi5T] - [Whi5P] + K_{sbf}}$$

Cell cycle components represented by the model:

**Variable**	**Cell cycle component**
Cln3	Total Cln3 bound CDK
Cln1/2	Total Cln1 or Cln2 bound CDK
Clb5/6	Total Clb5 or Clb6 bound CDK
Sic1	Total Sic1, includes free Sic1, Sic1 bound to Clb5 or Clb6 CDK, phosphorylated or unphosphorylated Sic1
Sic1P_1 to Sic1P_9	Sic1 phosphorylated 1 to 9 times
Sic1P	Total Sic1 that are phosphorylated at least 6 times, these Sic1 species have larger degradation rate compared to the rest of Sic1
Sic1PT	Total phosphorylated Sic1
$\text{Clb5/6}_\text{active}$	Clb5 or Clb6 CDK not bound by Sic1
C5S	Clb5 or Clb6 CDK Sic1 complex
Whi5T	Total Whi5
Whi5P	Phosphorylated Whi5
MBFT	Total MBF
MBFP	Phosphorylated MBF (active)
SBFT	Total SBF
SBFA	Activated SBF (not bound by Whi5)

Sic1m	Total Sic1 reporter (does not inhibit Clb5 or Clb6, but has)
Sic1mP$_1$ to Sic1mP$_9$	Sic1 reporter phosphorylated 1 to 9 times
Sic1mP	Total Sic1 reporter that are phosphorylated at least 6 times, these Sic1 reporter species have larger degradation rate compared to the rest of Sic1 reporters
Sic1mPT	Total phosphorylated Sic1 reporter

Experimental results used to constrain the model:
1) Protein amounts (Cross et al., 2002; Lu et al., 2007)
2) *CLN2* and *CLB5* promoter activity in *WT*, *mbp1Δ* and *swi4Δ* (Supplementary Table S7)
3) Sic1 and Sic1 reporter half lives in *WT* and other mutants (Supplementary Table S1, and S3)

Parameters:
Some parameters were derived from published experimental results, others were found first by using Latin Hypercube sampling followed by changing by hand to improve the fit.
Experimental results used for deriving parameters:
1) half lives of Cln3 (Tyers et al., 1992; Yaglom et al., 1995), Cln2 (Lanker et al., 1996; Schneider et al., 1998; Schneider et al., 2004; Salama et al., 1994; Deshaies et al., 1995; Willems et al., 1996; Germain et al., 1997), Clb5 (Germain et al., 1997; Seufert et al., 1995; Irniger and Nasmyth, 1997) , and Sic1(Nash et al., 2001; Verma et al., 2001)
2) k_{on} and k_{off} for Sic1 and its substrate (Verma et al., 2001; Barberis et al., 2005a; Barberis et al., 2005b; Mendenhall, 1993)
3) Multisite phosphorylation of Sic1 (Nash et al., 2001).

Parameters and initial values for *WT*.

WT		
$k_{n3} = 8.6\times10^{-4}\mu M min^{-1}$,	$d_{n3} = 0.15 min^{-1}$,	
$k_{n2,1} = 1\times10^{-6}\mu M min^{-1}$,	$k_{n2,sbf} = 3.42\times10^{-2}\mu M min^{-1}$,	$K_{n2,sbf} = 8.37\times10^{-2}$,
$k_{n2,mbf} = 2.43\times10^{-2}\mu M min^{-1}$,	$K_{n2,mbf} = 4.88\times10^{-1}$,	$d_{n2} = 1.3\times10^{-1} min^{-1}$,
$k_{b5,1} = 1\times10^{-6}\mu M min^{-1}$,	$k_{b5,sbf} = 2.49\times10^{-3}\mu M min^{-1}$,	$K_{b5,sbf} = 3.33\times10^{-1}$,
$k_{b5,mbf} = 2.57\times10^{-3}\mu M min^{-1}$,	$K_{b5,mbf} = 3.01\times10^{-1}$,	$d_{b5} = 3.5\times10^{-2} min^{-1}$,
$k_{c1} = 2\times10^{-3}\mu M min^{-1}$,	$K_{c1} = 1.05\times10^{-2}\mu M$	
$d_{c1,1} = 1.54\times10^{-2} min^{-1}$,	$d_{c1,2} = 2\times10^{-1} min^{-1}$,	
$k_{as} = 100\mu M^{-1} min^{-1}$,	$k_{di} = 2.4\times10^{-1} min^{-1}$,	
$k_{c1m} = 6\times10^{-3}\mu M min^{-1}$,	$d_{c1m,1} = 1.2\times10^{-2} min^{-1}$,	$d_{c1m,2} = 1.3\times10^{-1} min^{-1}$,

$\epsilon_{whi5,n3} = 532\mu M^{-1}$,	$\epsilon_{whi5,n2} = 30.9\mu M^{-1}$,	$\epsilon_{whi5,b5} = 28.2\mu M^{-1}$,
$\epsilon_{mbf,n3} = 65.2\mu M^{-1}$,	$\epsilon_{mbf,n2} = 16.8\mu M^{-1}$,	$\epsilon_{mbf,b5} = 13.0\mu M^{-1}$,
$\epsilon_{sic1m,n2} = 3.51\mu M^{-1}min^{-1}$, $\epsilon_{sic1m,b5} = 171\mu M^{-1}min^{-1}$,		
$\epsilon_{sic1,n2} = 2.91\mu M^{-1}min^{-1}$, $\epsilon_{sic1,b5} = 175\mu M^{-1}min^{-1}$,		
$k_{dp} = 5\times10^{-1}min^{-1}$,	$n_{whi5} = 4.28$,	$n_{mbf} = 4.03$,
[Whi5T]=3, [MBFT]=1,		[SBFT]=1,
$K_{sbf} = 4.83\times10^{-3}$,	$[Cln2(0)] = 7.69\times10^{-6}\mu M$,	$[Clb5(0)] = 2.86\times10^{-6}\mu M$,
$[Sic1(0)] = 0.03\mu M$, $[Sic1m(0)] = 0.13\mu M$		

Parameters and initial values for the results shown in Figure 2G and Figure S1G.

Strains	Parameters
cln2Δ	$k_{n2,1} = 2.5\times10^{-7}\mu Mmin^{-1}$, $k_{n2,sbf} = 8.55\times10^{-3}\mu Mmin^{-1}$ $k_{n2,mbf} = 6.08\times10^{-3}\mu Mmin^{-1}$, $[Cln2(0)] = 1.92\times10^{-6}\mu M$
clb5Δ	$k_{b5,1} = 2.5\times10^{-7}\mu Mmin^{-1}$, $k_{b5,sbf} = 6.23\times10^{-4}\mu Mmin^{-1}$, $k_{b5,mbf} = 6.43\times10^{-4}\mu Mmin^{-1}$, $[Clb5(0)] = 7.15\times10^{-7}\mu M$
mbp1Δ	[MBFT]=0
swi4Δ	[SBFT]=0
whi5Δ	[Whi5T]=0
clb6Δ	$k_{b5,1} = 7.5\times10^{-7}\mu Mmin^{-1}$, $k_{b5,sbf} = 1.87\times10^{-3}\mu Mmin^{-1}$, $k_{b5,mbf} = 1.93\times10^{-3}\mu Mmin^{-1}$, $[Clb5(0)] = 2.15\times10^{-6}\mu M$
cln1Δ cln2Δ	$k_{n2,1} = 0\mu Mmin^{-1}, k_{n2,sbf} = 0\mu Mmin^{-1}, k_{n2,mbf} = 0\mu Mmin^{-1}$, $[Cln2(0)] = 0\mu M$
clb5Δ clb6Δ	$k_{b5,1} = 0\mu Mmin^{-1}, k_{b5,sbf} = 0\mu Mmin^{-1}, k_{b5,mbf} = 0\mu Mmin^{-1}$, $[Clb5(0)] = 0\mu M$
swi6Δ	[MBFT]=0, [SBFT]=1/3

Parameters and initial values for mutants are the same as wild type except for those specified.

Simulation:

Each data point is an independent random realization of the model. Noise intrinsic to the system is modeled by simulating the system stochastically using the CLE framework (Gillespie, 2000; Gillespie 2007). Briefly, when simulating the system without noise, for each variable y_i with rate of change $\frac{dy_i}{dt} = f_i(\bar{y})$, at each time increment dt, $y_i(t + dt) = y_i(t) + f_i(\bar{y})dt$. When simulating with noise, a random term is added at each dt such that $y_i(t + dt) = y_i(t) + f_i(\bar{y})dt + \sqrt{f_i(\bar{y})dt}\mathcal{N}(0,1)$ where $\mathcal{N}(0,1)$ is a

random number drawn from a standard normal distribution. If the rate of change has both positive and negative terms i.e. $\frac{dy_i}{dt} = f_i(\bar{y}) - g_i(\bar{y})$ where $f_i(\bar{y}) \geq 0,\ g_i(\bar{y}) \geq 0$, then at each dt, $y_i(t+dt) = y_i(t) + (f_i(\bar{y}) - g_i(\bar{y}))dt + \sqrt{(f_i(\bar{y}) + g_i(\bar{y}))dt}\mathcal{N}(0,1)$ since variances are always added. To simulate extrinsic noise, for each simulation, every parameter in the model is sampled from a Gaussian distribution with mean equals to the listed value and a standard deviation equals to 20% of the mean. The half-life of Sic1 is fitted using an exponential function (as described in the "Half-life analysis" section). The start of the fit range is the point in the decay curve where Sic1decays the fastest and the end of fit range is the point where Sic1 drop below 25% of the amount at the fit range start point.

Supplemental References

Barberis, M., De Gioia, L., Ruzzene, M., Sarno, S., Coccetti, P., Fantucci, P., Vanoni, M., and Alberghina, L. (2005a). The yeast cyclin-dependent kinase inhibitor Sic1 and mammalian p27Kip1 are functional homologues with a structurally conserved inhibitory domain. Biochem. J *387*, 639–647.

Barberis, M., Pagano, M.A., Gioia, L.D., Marin, O., Vanoni, M., Pinna, L.A., and Alberghina, L. (2005b). CK2 regulates in vitro the activity of the yeast cyclin-dependent kinase inhibitor Sic1. Biochem. Biophys. Res. Commun *336*, 1040–1048.

Cross, F.R., Archambault, V., Miller, M., and Klovstad, M. (2002). Testing a mathematical model of the yeast cell cycle. Mol. Biol. Cell *13*, 52–70.

Deshaies, R.J., Chau, V., and Kirschner, M. (1995). Ubiquitination of the G1 cyclin Cln2p by a Cdc34p-dependent pathway. EMBO J *14*, 303–312.

Germain, D., Hendley, J., and Futcher, B. (1997). DNA damage inhibits proteolysis of the B-type cyclin Clb5 in S. cerevisiae. J. Cell. Sci *110 (Pt 15)*, 1813–1820.

Gillespie, D.T. (2000). The chemical Langevin equation. J. Chem. Phys. *113*, 297–306.

Gillespie, D.T. (2007). Stochastic Simulation of Chemical Kinetics. Annu. Rev. Phys. Chem. *58*, 35-55

Irniger, S., and Nasmyth, K. (1997). The anaphase-promoting complex is required in G1

arrested yeast cells to inhibit B-type cyclin accumulation and to prevent uncontrolled entry into S-phase. J. Cell. Sci *110 (Pt 13)*, 1523–1531.

Lanker, S., Valdivieso, M.H., and Wittenberg, C. (1996). Rapid degradation of the G1 cyclin Cln2 induced by CDK-dependent phosphorylation. Science *271*, 1597–1601.

Lu, P., Vogel, C., Wang, R., Yao, X., and Marcotte, E.M. (2007). Absolute protein expression profiling estimates the relative contributions of transcriptional and translational regulation. Nat. Biotechnol *25*, 117–124.

Mendenhall, M.D. (1993). An inhibitor of p34CDC28 protein kinase activity from Saccharomyces cerevisiae. Science *259*, 216–219.

Nash, P., Tang, X., Orlicky, S., Chen, Q., Gertler, F.B., Mendenhall, M.D., Sicheri, F., Pawson, T., and Tyers, M. (2001). Multisite phosphorylation of a CDK inhibitor sets a threshold for the onset of DNA replication. Nature *414*, 514–521.

Salama, S.R., Hendricks, K.B., and Thorner, J. (1994). G1 cyclin degradation: the PEST motif of yeast Cln2 is necessary, but not sufficient, for rapid protein turnover. Mol. Cell. Biol *14*, 7953–7966.

Schneider, B.L., Patton, E.E., Lanker, S., Mendenhall, M.D., Wittenberg, C., Futcher, B., and Tyers, M. (1998). Yeast G1 cyclins are unstable in G1 phase. Nature *395*, 86–89.

Schneider, B.L., Zhang, J., Markwardt, J., Tokiwa, G., Volpe, T., Honey, S., and Futcher, B. (2004). Growth rate and cell size modulate the synthesis of, and requirement for, G1-phase cyclins at start. Mol. Cell. Biol *24*, 10802–10813.

Seufert, W., Futcher, B., and Jentsch, S. (1995). Role of a ubiquitin-conjugating enzyme in degradation of S- and M-phase cyclins. Nature *373*, 78–81.

Tyers, M., Tokiwa, G., Nash, R., and Futcher, B. (1992). The Cln3-Cdc28 kinase complex of S. cerevisiae is regulated by proteolysis and phosphorylation. EMBO J *11*, 1773–1784.

Verma, R., McDonald, H., Yates, J.R., and Deshaies, R.J. (2001). Selective degradation of ubiquitinated Sic1 by purified 26S proteasome yields active S phase cyclin-Cdk. Mol. Cell *8*, 439–448.

Willems, A.R., Lanker, S., Patton, E.E., Craig, K.L., Nason, T.F., Mathias, N., Kobayashi, R., Wittenberg, C., and Tyers, M. (1996). Cdc53 targets phosphorylated G1 cyclins for degradation by the ubiquitin proteolytic pathway. Cell *86*, 453–463.

Yaglom, J., Linskens, M.H., Sadis, S., Rubin, D.M., Futcher, B., and Finley, D. (1995).

p34Cdc28-mediated control of Cln3 cyclin degradation. Mol. Cell. Biol *15*, 731–741.
