# Supplementary material for: Design Principles of the Yeast G1/S Switch
Source: PLoS Biol. 2013 Oct 1;11(10):e1001673. doi: 10.1371/journal.pbio.1001673 (PMC3794861; doi:10.1371/journal.pbio.1001673)
Supplement: Text S3 — In silico evolution. (PDF) [file pbio.1001673.s016.pdf]

Supplemental Text S3.

In silico evolution

Our simulations employ an ODE model of the switch (Figure 6A) based on individual reactions between the components, Kinase1, Kinase2, and Inhibitor, which are analogous to Cln1/2-Cdk1, Clb5/6-Cdk1, and Sic1 in the yeast G1/S switch. We modeled Inhibitor with six phosphosites, all of which required to be phosphorylated for its rapid degradation. Multisite phosphorylation of Inhibitor was assumed to occur in an ordered fashion, from site #1 through site #6. The reaction set is given below.

```
# KINASE1 and KINASE2 synthesis
kinase1 → kinase1 + KINASE1, Hill(t, ks1, Ks1, ns1)
kinase2 → kinase2 + KINASE2, Hill(t, ks2, Ks2, ns2)

# KINASE1 AND KINASE2 degradation
KINASE1 → Ø, kd1
KINASE2 → Ø, kd2

# INHIBITOR synthesis
inhibitor → inhibitor + INHIBITORp0, ks3

# Phosphorylation of free INHIBITOR by KINASE1
INHIBITORpi + KINASE1 → KINASE1 + INHIBITORpi+1, k1,i

# Phosphorylation of INHIBITOR-KINASE2 complex by KINASE1
INH_K2pi + KINASE1 → KINASE1 + INH_K2pi+1, k1,i

# Phosphorylation of free INHIBITOR by KINASE2
INHIBITORpi + KINASE2 → KINASE2 + INHIBITORpi+1, k2,i

# Phosphorylation of INHIBITOR-KINASE2 complex by KINASE2
INH_K2pi + KINASE2 → KINASE2 + INH_K2pi+1, k2,i

# Dephosphorylation of free INHIBITOR
INHIBITORpi+1 → INHIBITORpi, k3

# Dephosphorylation of INHIBITOR-KINASE2 complex
INH_K2pi+1 → INH_K2pi, k3

# Sequestration of free KINASE2 by free INHIBITOR
INHIBITORpj + KINASE2 → INH_K2pj, k4

# Dissociation of INHIBITOR-KINASE2
INH_K2pj → INHIBITORpj + KINASE2, k5

# Degradation of free INHIBITOR
```

$INHIBITORp_j \rightarrow \emptyset,\ k_{6,i}$

Degradation of INHIBITOR-KINASE2 complex to free KINASE2
$INH\_K2p_j \rightarrow KINASE2,\ k_{6,i}$

Degradation of INH_K2 to free INHIBITOR
$INH\_K2p_j \rightarrow INHIBITORp_j,\ k_{d2}$

Here, lowercase symbols, kinase1, kinase2, and inhibitor denote genes; whereas uppercase symbols, KINASE1, KINASE2, and $INHIBITORp_i$ denote proteins. INH_K2p_i represents the Inhibitor-Kinase2 complex. Rate of each reaction is given after the comma. The reactions for KINASE1 and KINASE2 synthesis employ Hill functions to produce sigmoidal curves. These reactions are implemented as a function of time. The reaction rate is calculated using the function $\mathrm{Hill}(t,k,K,n) = k\frac{t^n}{K^n+t^n}$, where t represents time in seconds. The indices, i and j are integers denoting the number of phosphorylated sites ($0 \le i \le 5$ and $0 \le j \le 6$). In the 1P model, i=0 and j=0, 1. We used molecule counts in the simulations (not concentrations), and chose $k_{s1}=k_{s2}=160$, $k_{d1}=k_{d2}=0.08$, $K_{s1}=K_{s2}=10$, $n_{s1}=n_{s2}=4$, $k_{s3}=23.1$, $k_3=0.5$, $k_4=0.5$, $k_5=0.24$, $k_{6,i<6}=0.0154$, $k_{6,6}=0.17$. Constants, k_{s1}, k_{s2}, k_{d1}, k_{d2}, K_{s1}, K_{s2}, n_{s1}, and n_{s2} are subject to extrinsic noise (to simulate cell to cell variability). Each time a copy of the circuit is run, the specified reaction rate is multiplied by $1+\rho$, where ρ is a random number uniformly distributed on $(-x, x)$. x is chosen as 0.2 for k_{s1}, k_{s2}, K_{s1}, and K_{s2}; and 0.7 for n_{s1} and n_{s2}. The phosphorylation rates, $k_{1,i}$, and $k_{2,i}$ are real numbers between $[10^{-10}, 0.1]$ and are subject to mutations. At the beginning of the simulation, they are assigned random numbers from a log-uniform distribution on $[10^{-6}, 0.1]$. Dephosphorylation was assumed to occur at a constant rate. Mutations are modeled by multiplying the reaction rates by a random number uniformly distributed on (0, 2]. Mutation rate per reaction rate per generation was chosen as 0.3. Mutations are carried from one generation to the next, unlike the extrinsic noise, which is not heritable. $k_{1,i}= 0$ in the double-negative feedback loop simulations, and $k_{2,i}= 0$ in the linear circuit.

We define timing of the Kinase2 activation as the time the number of Kinase2 exceeds the number of Inhibitor. We take sharpness as the slope of the free Kinase2 curve at its half-maximum level.

Optimization by *in silico* Evolution

The algorithm resembles evolution by natural selection, and works as follows.

1. **Initialization**. N=1000 copies of the network is generated. Phosphorylation rates of each copy (realization) are assigned random rates as discussed above. Initial protein counts are all zero except Inhibitor=1500. All genes have exactly one copy each.

2. **Scoring**. Reaction rates that are subject to extrinsic noise are modified as described above. (In this set, only Kinase1 and Kinase2 production/degradation are subject to noise.) Then, the network is run, and a fitness score is calculated based on the output. Fitness score for timing is

$$f_s = (t_{\mathrm{activation}} - t_{\mathrm{desired}})^2.$$

   Here t_{desired} denotes desired activation time for Kinase2. Sharpness score is simply the sharpness of the free Kinase2 curve as defined above. Lower scores mean higher fitness.

3. **Elimination and duplication.** Half of the population with lower fitness is eliminated. The rest is duplicated to make up the deficit.

4. **Mutation.** Duplicates are mutated as described above. Mutations only affect phosphorylation rates (*i.e.* catalytic efficiencies).

Steps 2-4 are repeated for m=1000 generations. Longer runs were not observed to change the final distributions of the phosphorylation rates qualitatively.

Simulations were written in C++ for distributed-memory parallel processing using OpenMPI (Gabriel et al., 2004). Sundials libraries were used for integration (Hindmarsh et al., 2005). The source code is available on request.

Supplemental References

Gabriel, E., Fagg, G.E., Bosilca, G., Angskun, T., Dongarra, J.J., Squyres, J.M., Sahay, V., Kambadur, P., Barrett, B., Lumsdaine, A., et al. (2004). Open MPI: Goals, concept, and design of a next generation MPI implementation. In In Proceedings, 11th European PVM/MPI Users' Group Meeting, pp. 97–104.

Hindmarsh, A.C., Brown, P.N., Grant, K.E., Lee, S.L., Serban, R., Shumaker, D.E., and Woodward, C.S. (2005). SUNDIALS: Suite of nonlinear and differential/algebraic equation solvers. ACM Trans. Math. Softw. *31*, 363–396.
